# Boosting ovarian cancer immunotherapy via discoidin domain receptor 2 blockade and CD28 x mucin 1 bispecific antibodies

**DOI:** 10.1186/s12916-026-05183-2

**Published:** 2026-08-24

**Authors:** Martina Kunkl, Silvia Malatesta, Flavia Ottavi, Ilenia Masi, Manolo Sambucci, Virginia Vigiano Benedetti, Maria Egle De Stefano, Martina Leopizzi, Maria Teresa Fiorillo, Marco Rossi, Francesco Mura, Anacleto Proietti, Enrica Martinelli, Camilla Nero, Anna Fagotti, Giovanna Borsellino, Luca Battistini, Valerio Licursi, Francesco Spallotta, Laura Rosanò, Loretta Tuosto

**Affiliations:** 1 Department of Biology and Biotechnologies “Charles Darwin”, Sapienza University of Rome, Rome, Italy; 2 Istituto Pasteur Italia – Fondazione Cenci Bolognetti, Sapienza University of Rome, Rome, Italy; 3 Institute of Molecular Biology and Pathology (IBPM), CNR, Rome, Italy; 4 Department of Internal Medicine and Medical Specialties – DIMI, University of Genova, Genova, Italy; 5 LIFE Institute for Research and Health Care - Santa Lucia IRCCS, Rome, Italy; 6 Department of Basic and Applied Sciences for Engineering, Sapienza University of Rome, Rome, Italy; 7 Research Center for Nanotechnology applied to Engineering, Sapienza University of Rome, Rome, Italy; 8 Università Cattolica del Sacro Cuore, Rome, Italy; 9 Unit of Oncological Gynaecology, Department of Woman, Child and Public Health, Fondazione Policlinico Universitario Agostino Gemelli IRCCS, Rome, Italy

**Keywords:** Serous Ovarian Carcinoma, DDR2, Tumour-specific T lymphocytes, Transcriptomics, Immunotherapy, Bispecific antibodies

## Abstract

**Background:**

Serous ovarian carcinoma (SOC) is a highly aggressive gynaecological malignancy with frequent relapse and the development of chemoresistance. A major feature of SOC tumour microenvironment is the accumulation of dense collagen fibres, which promote tumour progression and immune evasion. Discoidin domain receptor 2 (DDR2), a collagen-binding receptor tyrosine kinase activated by fibrillar collagens, mediates extracellular matrix-associated signalling involved in tumour progression and immune exclusion. However, its role in limiting antitumour immunity and contributing to immunotherapy resistance in SOC remains poorly understood.

**Methods:**

We investigated the impact of DDR2 blockade on SOC-specific T cell activity and on the efficacy of recombinant bispecific antibodies (BsAbs) targeting CD28 and mucin 1 (MUC1). SOC-specific T cells were generated from healthy donor peripheral blood mononuclear cells and assessed for recruitment, activation, and cytotoxicity against low-grade (OVCA433) and high-grade (OVCAR3) SOC cell lines under different treatment conditions.

**Results:**

DDR2 inhibition enhanced SOC-specific T cell responses by remodelling the collagen matrix, favouring their recruitment, activation and cytotoxic activity against both low- and high-grade SOC cells. Moreover, combined treatment with DDR2 blockade and CD28xMUC1 BsAbs significantly increased the recruitment of SOC-specific T cells, granzyme B release, caspase-3 activation, and cytokine production.

**Conclusions:**

DDR2 blockade mitigates collagen-mediated immune exclusion and enhances the efficacy of CD28xMUC1 BsAb-based immunotherapy in SOC models. These findings provide a mechanistic rationale for further investigation of combined DDR2 targeting and CD28×MUC1 costimulatory strategies to improve ovarian cancer immunotherapy.

## Background

Serous ovarian carcinoma (SOC) is among the most aggressive gynaecological tumours and is associated with high morbidity and mortality rates [1]. SOC is classified into low-grade (LG-SOC), accounting for approximately 10% of cases, and high-grade (HG-SOC), which represents 60-80% of epithelial ovarian cancers and is the sixth leading cause of cancer-related death in women [2]. Although most patients initially respond to chemotherapy, approximately 70% experience relapses and ultimately develop chemoresistance [3]. Consequently, substantial efforts have focused on identifying more targeted therapeutic approaches, including strategies directed at the tumour microenvironment (TME), to improve treatment efficacy while minimising the toxicity associated with conventional chemotherapy [4].

The SOC TME is characterised by extensive extracellular matrix (ECM) remodelling, particularly the accumulation of dense fibrillar collagens that contribute to tumour progression and immune evasion [5]. Tumour and stromal cells secrete factors that induce the differentiation of stromal fibroblasts into cancer-associated fibroblasts (CAFs), which in turn produce extracellular matrix components, particularly collagen type I (Col1) [6]. Progressive collagen remodelling generates dense, aligned, and reticulated collagen fibres that increase tumour stiffness, support malignant cell invasion, and establish a stromal barrier limiting the infiltration of tumour-specific T cells [6]. Beyond their structural role, collagens actively regulate tumour progression by promoting metastasis, modulating cellular signalling, and contributing to local immunosuppression [7].

Among the principal mediators of collagen-dependent signalling are discoidin domain receptors (DDR) 1 and 2, collagen-binding receptor tyrosine kinases (RTKs) activated upon interaction with fibrillar collagens within the ECM [8]. Activation of DDRs by collagen engagement triggers multiple signalling pathways involved in tumour cell proliferation, chemoresistance, epithelial-to-mesenchymal transition (EMT), and suppression of antitumour immunity [9]. In ovarian cancer, DDR2 expression is significantly increased relative to normal ovarian tissues and positively correlates with tumour stage and peritoneal metastasis, as well as to therapy resistance [10,11].

Beyond its role in tumour progression, DDR2 has increasingly emerged as a regulator of tumour immune exclusion. Elevated DDR2 expression has been associated with impaired immune infiltration and poor clinical outcomes across multiple tumour types [12,13]. Furthermore, recent evidence indicates that DDR2 inhibition can enhance the efficacy of immune checkpoint blockade in several solid tumours, supporting the concept that collagen-mediated signalling contributes to resistance to antitumour immunity [14]. However, whether DDR2 limits SOC-specific T cell responses and contributes to resistance to ovarian cancer immunotherapy remains poorly understood.

Despite evidences that SOC exhibits an immunoreactive phenotype characterised by tumour-infiltrating lymphocytes (TILs) directed against multiple tumour-associated antigens (TAAs), clinical responses to immune checkpoint blockade have remained modest [15]. These observations suggest that additional mechanisms within the TME, including defective T-cell costimulation and stromal immune exclusion, may limit effective antitumour immunity. In this context, bispecific antibodies (BsAbs) targeting T-cell costimulatory pathways have emerged as promising next-generation immunotherapeutic strategies. CD28, a key costimulatory receptor required for full T cell activation and prevention of anergy, represents an attractive therapeutic target [16]. BsAbs targeting CD28 and TAAs selectively provide costimulatory signals to tumour-reactive T cells, thereby enhancing T-cell cytotoxicity, persistence, and immunological memory while potentially limiting nonspecific systemic activation [17]. Importantly, recent preclinical studies have demonstrated encouraging safety and efficacy profiles for CD28xTAA-targeted BsAbs, particularly when combined with PD-1 blockade to sustain antitumour T-cell responses [18].

Based on these observations, we investigated whether targeting DDR2 could enhance the activation and cytotoxic function of SOC-specific T cells and improve responsiveness to CD28×mucin 1 (MUC1) BsAb-based immunotherapy. Specifically, we evaluated whether DDR2 inhibition could overcome collagen-mediated immune suppression and potentiate tumour-specific T-cell responses within collagen-rich ovarian cancer microenvironments. Our findings provide a mechanistic rationale for further investigation of combined DDR2 blockade and CD28×MUC1 BsAbs as a potential immunotherapeutic strategy for ovarian cancer, warranting future validation in relevant *in vivo* models.

## Methods

### Cells, antibodies and reagents

The human ovarian serous adenocarcinoma cell lines OVCA433 (RRID:CVCL_0475) and OVCAR3 (RRID: CVCL_0465) (ATCC, Manassas, VA) were cultured in RPMI-1640 supplemented with 10% FBS, L-glutamine, penicillin and streptomycin (Euroclone, UK). Peripheral blood mononuclear cells (PBMCs) were isolated from buffy coats of anonymous healthy donors (HD) (San Camillo Forlanini Hospital, Rome, Italy) and cultured in RPMI-1640 containing 5% human serum, L-glutamine, penicillin and streptomycin (Euroclone). HD signed the informed consent in accordance with national legislation and the World Medical Association’s Code of Ethics for Medical Research Involving Human Subjects (Declaration of Helsinki).

The following antibodies and reagents were used: mouse anti-collagen type I (COL1A) (#sc-59772, Santa Cruz Biotechnology, USA); anti-mouse HRP (#PAM008, SciTek Laboratories, USA); anti-human MUC1 (CD227)-PE (#130-120-142 Miltenyi Biotec, Italy); anti-human CD28-PE (#561793, BD Biosciences, Italy); mouse anti-human CD8 (#300902, Biolegend, USA); anti-human CD4-FITC (#21270043), anti-human CD3-PE (#21620034), anti-human CD8-APC (#21270086, ImmunoTools, Germany); rabbit anti-human CD3 (#AB16669, Abcam, UK); mouse anti-human CD3 clone LN10 (#PA0122, Leica Biosystems, Italy); goat anti-mouse Alexa Fluor 594 (#A11020), goat anti-rabbit Alexa Fluor 594 (#A11072), goat anti-mouse Alexa Fluor 488 (#A11070), goat anti-rabbit Alexa Fluor 488 (#A11070), goat anti-mouse Alexa Fluor 647 (#A21245), phalloidin-633 (#A22284), rabbit anti-DDR2 (#PA5-27752), 4′,6′-diamidino-2-phenykindole (DAPI) (#D1306, Thermo Fisher Scientific, Italy); rabbit anti-phospho-DDR1/DDR2 (pDDR2) (#MAB253882), anti-active human caspase-3 (#AF835, R&D Systems, USA); rat tail type 1 collagen (Col1) (#354236, Corning, USA); WRG-28 (#HY-114169, MedChemExpress, Sweden); dimethyl sulfoxide (DMSO) (#D-5879, Merck, Italy); human recombinant interleukin-2 (IL-2, #130-097-744) and IL-7 (#130-093-937, Miltenyi Biotec); rabbit anti-human granzyme B (#17215S, Cell Signalling, USA); mouse anti-GAPDH (#60004-1-IG, Proteintech, USA); CD28 x MUC1 (Tandem Fab) BsAbs (#BSFAB-H236, Creative Biolabs, USA); human Fab2 IgG (#NBP1-96779, Novus Biologicals, Bio-Techne, Italy); pembrolizumab (anti-PD1, #A2005, Selleck Biotechnology GmbH, Cologne); Cyto3D Live–Dead assay kit (#SKU:BM01, TheWell Bioscience, Inc., USA); DAPI (#1351303, Bio-Rad Laboratories, Italy); protease inhibitor MIX M (#3910201, SERVA, Germany); Mini-PROTEAN® TGX™ Precast Protein Gels (#4561034), DC protein assay (#5000115), Clarity Western ECL Substrates (#1705061, Bio-Rad Laboratories, Italy).

### Generation of SOC-specific T cell lines

OVCA433-specific and OVCAR3-specific T cell lines (TOVC) were generated as previously described [19]. Briefly, 10^8^ PBMCs from HD were cultured with 5 ×107 irradiated (100 Gy) OVCA433 cells (PBMC : OVCA433 ratio = 2 : 1) or 10^7^ OVCAR3 cells (PBMC : OVCAR3 ratio = 10 : 1) in RPMI-1640 supplemented with 5% human serum. After 48 hours, human IL-2 (4 ng ml-1) and IL-7 (10 ng ml-1) were added to the cultures. After 7 days, T cell cultures were restimulated with irradiated OVCA433 or OVCAR3 (T : OVCA433 ratio = 2 : 1; T : OVCAR3 ratio = 10 : 1) and irradiated (60 Gy) autologous cryopreserved PBMCs (T : PBMC ratio = 2 :1) and cultured for further 7 days in the presence of IL-2 and IL-7. Gamma irradiations from a ^137^Cs source were carried out using the Gammacell Exactor 40 (Nordion, Istituto Superiore di Sanità, ISS, Rome, Italy) [20]. At the end of culture, dead cells were removed by using the EasySep™ Dead Cell Removal (Annexin V) Kit (#17899) and, where indicated, CD8-enriched TOVC cell lines were obtained by depleting CD4^+^ T cells with the EasySep™ Human CD4 Positive Selection Kit II (#17852, STEMCELL Technologies, UK). The purity of the TOVC cell lines was evaluated by analysing the expression of CD3, CD4, CD8 and CD28 by flow cytometry (FACSCalibur, BD Biosciences, Italy).

### Flow cytometry analysis

To test the cytotoxic effects of WRG-28, TOVC, OVCA433 or OVCAR3 cells were cultured for 24 hours on Col1 (50 μg ml^-1^) in the presence of DMSO (1:500 dilution) as vehicle control, or 2 μM WRG-28. At the end of incubation, cells were stained with Cyto3D Live–Dead (LD) assay kit and analysed by flow cytometry (FACSCalibur, BD Biosciences, Italy).

For the multicolour analysis of activation markers, TOVC cells (10^6^) were stimulated for 24 hours with OVCA433 cells (2 ×105) grown overnight on 12-well plates coated with 50 μg ml^-1^ Col1 in the presence of DMSO (1:500 dilution) as vehicle control, or 2 μM WRG-28. At the end of incubation, cells were incubated at room temperature (RT) for 15 min with 30 μl of the antibody mixture listed in Additional file 1: Table 1, washed and analysed on a 6-laser CytoFLEX LX (Beckman Coulter). Data were analysed with FlowJo 10.8.1 (BD Biosciences).

### Elisa assays

TOVC cells (2.5×105) were cultured for 24 or 48 hours with adherent OVCA433 or OVCAR3 cells (5×104) grown overnight on 24-well plates coated with 50 μg ml^-1^ Col1, in the presence of DMSO (vehicle control), 2 μM WRG-28, isotype control human Fab2 IgG, the indicated concentrations of CD28xMUC1 BsAbs, or 10 μg ml^-1^ pembrolizumab (anti-PD1). At the end of incubation, secretion of cytokines in culture supernatants was measured by ELISA using human IFN-γ (#DY285), GM-CSF (#DY215), TNF-α (#DY-210), or IL-2 (#DY202, R&D Systems). The assays were performed in triplicate, and data were analysed on a CLARIOstar (BMG Labtech, Germany). The sensitivity of the assays was as follow: IFN-γ = 9.4 pg ml^-1^, GM-CSF, TNF-α and IL-2 = 15.6 pg ml^-1^.

### Western blotting

OVCA433 or OVCAR3 cells were detached by scraping, collected by centrifugation, and lysed in RIPA buffer [50 mM Tris-HCl (pH 7.5), 150 mM NaCl, 1% Nonidet P-40, 0.5% sodium deoxycholate (NaDoc), 0.1% SDS], together with protease and phosphatase inhibitors. Protein concentrations were determined using the DC Protein Assay (BIO-RAD, Italy). Cell lysates were resolved on 10% Mini-PROTEAN® TGX™ Precast Protein gels (BIO-RAD) and transferred to nitrocellulose membranes, followed by incubation with anti-pDDR1/2 (1:500 dilution), anti-DDR2 (1:500 dilution) and anti-GAPDH Abs (1:5000 dilution), and detected using horseradish peroxidase-conjugated secondary antibodies. Proteins were visualised by chemiluminescence using Clarity Western ECL Substrates with an Azure 300 imaging system (Azure Biosystems). Quantification analyses were performed using Fiji ImageJ software and represented the relative amounts as the ratio of each protein band to the loading control.

### Immunofluorescence and confocal microscopy analyses

TOVC cells (2 ×105) were stimulated for the indicated times with OVCA433, or OVCAR3 cells (4 ×104) adhered overnight on Col1-coated coverslips in the presence of DMSO (vehicle control), WRG-28, isotype control human Fab2 IgG, or CD28xMUC1 BsAbs. At the end of incubation, cells were fixed by 3% paraformaldehyde for 30 minutes at 4°C and permeabilized by 0.1% saponin in PBS containing 1% BSA for 1 hour at room temperature. Active caspase-3 (Casp3) staining was performed by using rabbit anti-human Casp3 (1:100 dilution) followed by goat anti-rabbit Alexa Flour 488 (1:150 dilution). Granzyme B (GzmB) and CD8 staining were performed by using rabbit anti-human GzmB Abs (1:100 dilution) followed by goat anti-rabbit Alexa Flour 488 Abs (1:150 dilution), and mouse anti-human CD8 (1:50 dilution) followed by goat anti-mouse Alexa Flour 594 Abs (1:100 dilution). Filamentous actin (F-actin) was stained by using phalloidin Alexa Fluor 633 (1:50 dilution). Nuclei were stained with DAPI (1:5000 dilution). All Abs and reagents were diluted in PBS containing 1% BSA and 0.1% saponin. Images of Casp3 nuclear localization were obtained with a 40x oil objective, Zeiss Apotome fluorescence microscope and Zen software. Fluorescence intensity analysis of Casp3 was performed at the single-cell level using Fiji ImageJ software. Individual tumour cells were identified based on DAPI staining, and regions of interest were manually delineated. Relative fluorescence intensity (RI) was quantified using identical acquisition and analysis settings across all conditions in at least 44 cells per condition from at least four microscopic fields. Images of GzmB levels in CD8^+^ T cells were obtained with a 40x oil objective Zeiss LSM900 confocal microscope. GzmB levels in CD8^+^ cells were quantified by measuring the Integrated Density (IntDen) using Fiji ImageJ software. To define GzmB positivity and calculate the percentage of GzmB^+^ cells, a threshold was established using the median RI value measured in CD8⁺ TOVC cells cultured with SOC on Col1 for each experimental condition.

For immunofluorescence analysis of DDR2 phosphorylation, OVCAR3 or OVCA433 cells were seeded on Col1-coated coverslips or gelatin-coated coverslips (10 µg ml^-1^) as negative control for 1 hour at 37 °C and treated with serum-free medium alone or with 2 µM WRG-28 for 6 hours. Cells were fixed by 4% paraformaldehyde for 10 min at room temperature, permeabilised by 0.2% Triton X-100, and blocked by 0.1 M glycine, 1% BSA, and 0.1% Tween-20 in PBS for 30 min at room temperature. Samples were stained with anti-pDDR2 (1:200 dilution) in 0.5% BSA in PBS overnight at 4 °C, followed by goat anti-rabbit Alexa Flour 488 for 1 hour at room temperature. F-actin was stained using phalloidin Alexa Fluor 633. Nuclei were stained with DAPI. Coverslips were mounted with Vectashield fluorescence mounting medium (Vector Laboratories) and imaged with an X-Light V3 confocal spinning-disk unit (CrestOptics) mounted on a Nikon Ti-E inverted motorised time-lapse microscope with integrated Perfect Focus System and DIC optics, equipped with a Kinetix CMOS camera (Photometrics) and a Celesta laser source (Lumencor).

### Real time PCR

Total RNA was extracted using TRizol^TM^ reagent (Thermo Fisher Scientific) from TOVC cells (10^6^), OVCA433 and OVCAR3 cells (2.5 × 10^5^), and reverse-transcribed into cDNA using Moloney murine leukaemia virus reverse transcriptase (Invitrogen). Real-time PCR was performed on a QuantStudio^TM^ 3 qPCR System (Applied Biosystem) using SensiFAST™ SYBR^®^ Hi-ROX One-Step Kit (Meridian Bioscience). The specific primers used were as follows: human DDR2, forward: 5’-AAACGACCCCAAACAACCCA-3’ and reverse: 5’- TGATCCCATCTATCCCTGCCA-3’; GAPDH, forward 5′-ACATCGCTCAGACACCATG-3’ and reverse 5′-TGTAGTTGAGGTCAATGAAGGGG-3′. The relative quantification was performed by using 2^−^ΔΔCt method.

### RNA-sequencing (RNA-SEQ)

OVCA433 and OVCAR3 cells (2.5 × 10^5^) were growth for 24 hours on 12-well plates coated with 50 μg ml^-1^ Col1, in the presence of DMSO (vehicle control), or 2 μM WRG-28. Total RNA extraction, mRNA enrichment, and poly-A RNA-Seq library preparation and sequencing were performed by the BGI Genomics Co., Ltd (Hong Kong, China). Four replicates for each experimental group were used. The final libraries for paired-end sequencing of 150 base pairs were carried out on DNBSEQ G400 PE150 platform (BGI). Reads quality was evaluated using FastQC (version 0.11.8, Babraham Institute Cambridge, UK) tool and then reads were mapped to the human NCBI GRCh38.p14.v2201 genome using [21] with the following settings: --sensitive --no-discordant --no-mixed -I 1 -X 1000 -p 8 --rna-strandness RF. The average alignment ratio of the sample comparison genome was 98.59% and a total of 18032 genes were detected.

To identify differentially expressed genes (DEGs) both gene level normalization and differential gene expression analysis were performed using Bioconductor R version 4.1.2 (R Core Team, 2015) package DESeq2 version 1.50.2 [22,23]. Differential expression was assessed by fitting negative binomial generalized linear models to gene-level count data, and statistical significance for each contrast was evaluated using the Wald test implemented in DESeq2. Resulting p values were adjusted for multiple testing using the Benjamini–Hochberg procedure to control the false discovery rate.

A total of 147 genes were identified as DEGs in OVCA433 and 49 in OVCAR3 using cut-offs of Fold change (FC) > 1.5 or < -1.5 and false discovery rate (FDR) < 0.05 (Additonal file 2: Table 2 and Additional file 3: Table 3). Volcano plots were created using Bioconductor R package EnhancedVolcano version 1.28.2. For visualization purpose the FDR values were transformed in -log10(FDR). DEGs were clustered by functional annotation in Gene Ontology (GO) and pathway enrichment analysis using Bioconductor R package clusterProfiler version 4.18.4 with annotation of Cellular Component of GO Database [24,25]. Enrichment significance was assessed using an over-representation analysis based on the hypergeometric test, and the resulting p values were adjusted for multiple comparisons using the Benjamini–Hochberg method to control the false discovery rate. Figures were obtained using the R environment with functions from ggplot2 package version 3.3.0.

### Analysis of TOVC cell recruitment into SOC-populated hydrogels

Hydrogels populated with OVCA433 or OVCAR3 cells were generated by adapting a previously published protocol [26]. Briefly, Col1 (9 mg ml⁻¹) was suspended in 0.02 N acetic acid, OVCA433 or OVCAR3 cells (10⁶) were added by gentle mixing in the presence or absence of WRG-28 (2 μM), and the pH was adjusted to 8 with 1 M NaOH. The mixture was distributed into transwell 12 well plates with 3 μm pores and incubated for 30 min in a vibration-free incubator to allow polymerisation. Following polymerisation, complete medium was added, and SOC-populated hydrogels were cultured for 24 h. TOVC cells (2 × 10⁶) were then seeded on top of the OVCA433- or OVCAR3-populated hydrogels and incubated for the indicated times with WRG-28 and/or CD28 x MUC1 bispecific antibodies (BsAbs). At the end of incubation, hydrogels were fixed by 4% formalin and embedded in paraffin. Sections (7 μm; n = 6) were obtained by MICROM HM315 microtome (International GMBH) and processed for immunohistochemistry and immunofluorescence. Immunohistochemistry analyses were performed by staining the sections with mouse anti-COL1A (1:100 dilution) followed by anti-mouse-HRP-conjugated Abs, using a MOTIC AE200 microscope (Motic, USA). Immunofluorescence analyses of T cell recruitment were performed by staining with rabbit anti-human CD3 (1:100 dilution) followed by goat anti-rabbit Alexa Fluor 488 Abs (1:200 dilution), and mouse anti-human pan-cytokeratin (PanCK) (1:250 dilution) followed by goat anti-mouse Alexa Fluor 594 Abs (1:200 dilution). Confocal imaging was performed using a Zeiss LSM900 microscope equipped with a 20x oil objective. The number of CD3^+^ cells was quantified across six sections (approximately 1.6 cm^2^), using Fiji ImageJ software.

### Scanning electron Microscopy

The organisation of collagen fibres in OVCA433-populated hydrogels was analysed using a Zeiss Auriga 405 Field Scanning Electron Microscopy. Hydrogel sections (7 µm) were mounted on a silicon wafer (Si) substrate, deparaffinised, and coated with a 10 nm-thick conductive chromium layer using a Quorum Q150T sputtering system. The images were acquired at magnifications of 10.00 and 3.00 kX.

Scanning electron microscopy micrographs acquired at 10,000× magnification were quantitatively analysed using Fiji ImageJ software. Prior to analysis, regions containing cells were manually excluded to ensure that only the fibrous scaffold architecture was evaluated. The remaining scaffold area was converted into binary images using the automatic Otsu thresholding algorithm to discriminate the fibrous phase from pore space. Identical thresholding and analysis parameters were applied to all images. The fraction of image area occupied by collagen fibres was quantified, yielding the percentage of scaffold material within the analysed region. Hydrogel porosity was subsequently estimated from binarized images according to the following equation: porosity (%) = 100 − area fraction (%). A total of 12 scanning electron microscopy micrographs obtained from three independent experiments were analysed for each condition. Data are presented as median values, and porosity measurements were used to compare structural differences in collagen network architecture among the different scaffold formulations and treatment conditions.

### Bioinformatic analyses of publicly available transcriptomic datasets

Bioinformatic analyses were performed using publicly available datasets from The Cancer Genome Atlas (TCGA) accessed through the cBioPortal platform (https://www.cbioportal.org/). The Ovarian Serous Cystadenocarcinoma dataset [27] was used to retrieve mRNA expression data. Expression levels for DDR2 and selected genes associated with extracellular matrix remodelling and immune regulation, including ACTA2, COL1A1, CD8A, and CD274, were extracted from cBioPortal. The downloaded data were subsequently analysed using GraphPad Prism version 10.0 (GraphPad Software, San Diego, CA, USA). Correlation analyses were performed using Spearman’s rank correlation coefficient, and scatter plots were generated to visualize pairwise gene expression relationships. Statistical significance was assessed using two-tailed p-values, with a threshold set at p < 0.05. Progression-free survival (PFS) analyses were performed using the Kaplan-Meier Plotter tool (KMplotte r; https://kmplot.com/analysis/). DDR2 expression was evaluated using the Affymetrix probe set 205168_at, while combined DDR2/MUC1 expression analyses were performed using the JetSet best probe selection available within the platform (including probe sets such as 205168_at for DDR2 and 225442_at for MUC1). Patients were stratified into high and low expression groups using the auto-selected best cut-off (percentile-based). The analysis was restricted to serous ovarian carcinoma patients, including all stages, with grade 1–2 tumours for the LG-SOC cohort and grade 3 for HG-SOC cohort. A follow-up threshold of 120 months was applied, and biased arrays were excluded using the platform’s quality control settings. Survival differences between groups were evaluated using the log-rank test, and hazard ratios (HRs) with 95% confidence intervals were reported. Data censoring was applied according to KMplotter default settings. mRNA expression above or below the median separates the cases into high- and low-expression levels. Hazard ratios (HR), 95% confidence intervals, and log-rank P are presented in the main plots.

### Patient Cohort and immunohistochemical analysis

This study included tissue samples from 5 patients diagnosed with HG-SOC who underwent surgical treatment. All samples were formalin-fixed and paraffin-embedded (FFPE) and retrieved from the archives of the institutional pathology department. Histopathological diagnoses were confirmed by experienced pathologists according to established criteria. Immunohistochemistry was performed on 4-µm sections from formalin-fixed, paraffin-embedded (FFPE) tissue blocks. Sections were deparaffinized in xylene and rehydrated through graded ethanol. Endogenous peroxidase activity was blocked with 3% hydrogen peroxide for 10 min at room temperature. Antigen retrieval was carried out using a pH 9 retrieval buffer under heat-induced conditions. For CD3, sections were subjected to antigen retrieval for 20 minutes, whereas for DDR2 antigen retrieval was performed for 40 minutes. After cooling to room temperature and washing in PBS, sections were incubated with anti-CD3 (clone LN10) and anti-DDR2 (1:200 dilution). Immunoreactivity was detected using a polymer-based detection system and visualized with 3,3′-diaminobenzidine (DAB). Slides were counterstained with haematoxylin, dehydrated, and mounted. Negative controls were obtained by omitting the primary antibody.

### Statistical analysis

Sample size was selected based on previous studies to ensure adequate statistical power. Data are presented as mean ± standard error of the mean (SEM), unless otherwise indicated. Statistical analyses were performed using GraphPad Prism version 10.0. For comparisons between two groups, a two-tailed Student’s t-test, was used for normally distributed data, whereas the Mann–Whitney U test was applied for non-parametric data. Comparisons among multiple groups were analysed using one-way ANOVA followed by Fisher’s least significant difference (LSD) post hoc test for multiple comparisons when parametric assumptions were met. Data distribution and variance were assessed prior to statistical analysis. A p value < 0.05 was considered statistically significant.

## Results

### Inhibition of DDR2 by WRG-28 enhances SOC-specific T cell responses

**Fig. 1:**
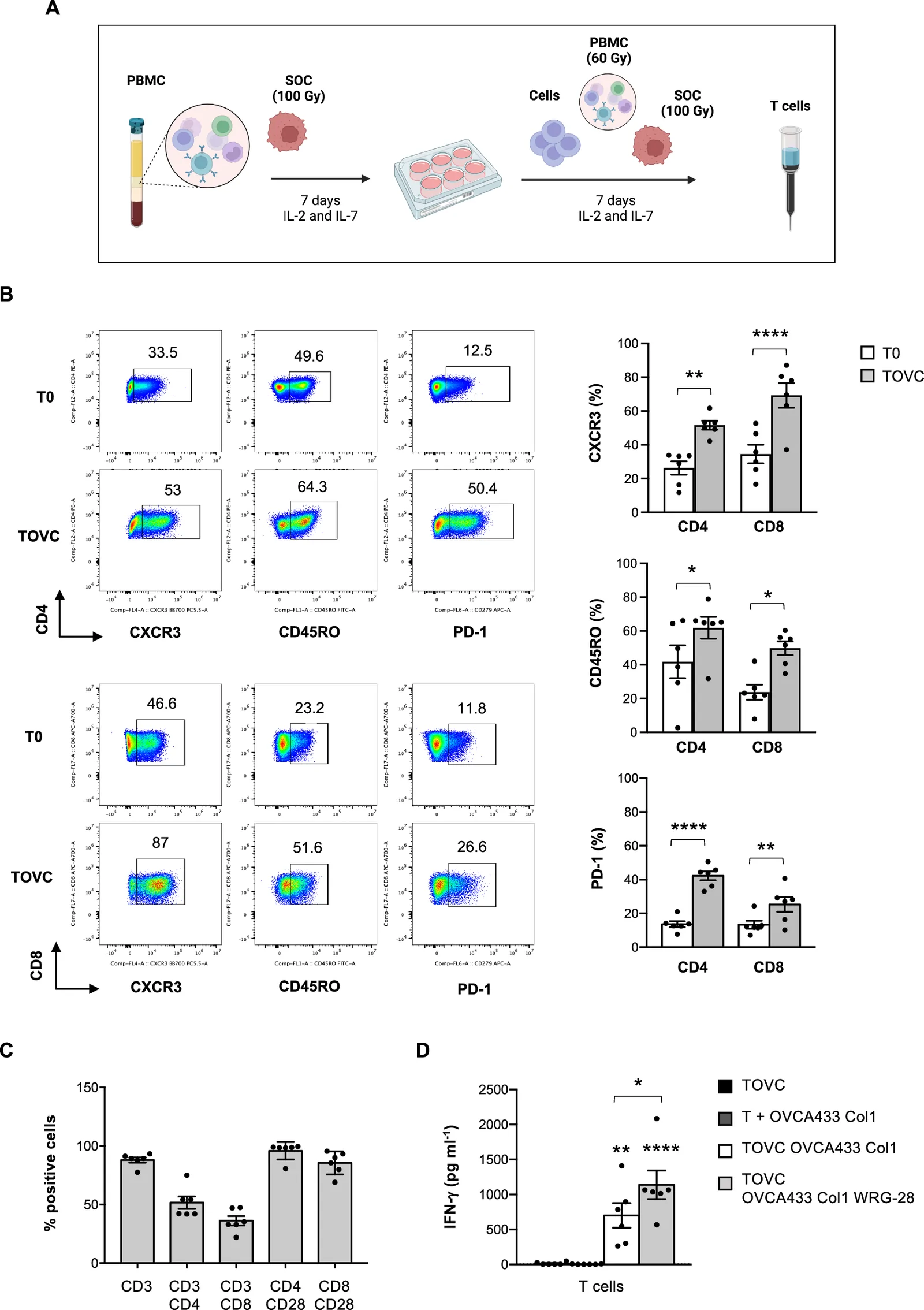
DDR2 inhibition enhances OVCA433-specific T-cell responses. (**A**) Schematic representation of SOC-specific T cell (TOVC) generation (created with BioRender; agreement number LB290UX50A). (**B, C**) Representative flow cytometry profiles and summary quantification of CD3, CD4, CD8, CD45RO, CXCR3, and PD-1 (CD279) expression in primary T-cell precursors (T0) and OVCA433-specific TOVC cell lines following two rounds of stimulation with OVCA433 cells. Data are presented as mean ± SEM from six independent TOVC cell lines (n = 6). (**D**) IFN-γ production in culture supernatants of TOVC cell lines stimulated for 24 hours with OVCA433 on Col1 in the presence of DMSO (vehicle control) or 2 μM WRG-28. IFN-γ levels were also measured in matched primary T-cell precursors stimulated under the same conditions. Data are presented as mean ± SEM from six independent experiments. Statistical significance was determined by one-way ANOVA with Fisher’s LSD post hoc test. * *p* < 0.05, ** *p* < 0.01, **** *p* < 0.0001

To assess the efficacy of inhibiting DDR2/Col1 interaction on SOC-specific T cell responses, we used the human ovarian serous adenocarcinoma cell line OVCA433 sharing most of the genetic and molecular features of LG-SOC, including cell-surface markers, making it a suitable surrogate model for SOC [28]. OVCA433 cells express DDR2 (Additional file 4: Fig. S1A), which is activated upon binding to Col1, as demonstrated by phosphorylation of tyrosine 740 (Additional file 4: Fig. S1B) [29]. OVCA433-specific T cell lines (TOVC) were generated by adapting a previously described protocol [19]. Peripheral blood mononuclear cells (PBMCs) from healthy donors (HD) were stimulated with irradiated OVCA433 cells (PBMC:OVCA433 ratio = 2:1) in the presence of recombinant human IL-2 and IL-7. After 7 days, T cells were restimulated for one week with irradiated OVCA433 cells and autologous PBMCs (T:OVCA433:PBMC ratio = 2:1:1), IL-2, and IL-7 (Fig. 1A). At the end of stimulation, we obtained TOVC cell lines (n = 6), showing 88% purity based on anti-CD3 staining, and comprising 51% CD3⁺CD4⁺ and 36% CD3⁺CD8⁺ T cells. CD28 expression was detected in both CD3⁺CD4⁺ (96%) and CD3⁺CD8⁺ T cells (86%). In addition, phenotypic analysis of activation markers associated with antigenic T-cell stimulation [4,30,31] revealed increased expression of CD45RO, CXCR3, and PD-1 on both CD4^+^ and CD8^+^ TOVC cells compared with primary T-cell precursors (T0) (Fig. 1B-C).

To evaluate the inhibitory effects of DDR2 inhibition on OVCA433-specific T cell responses, we performed a kinetic analysis by culturing TOVC cells with OVCA433 cells adhered overnight on Col1-coated plates in the presence of DMSO (vehicle control) or WRG-28, a selective allosteric inhibitor of the extracellular domain of DDR2 that binds to Col1 [32]. Treatment with WRG-28 significantly inhibited Col1-induced DDR2 phosphorylation (Additional file 4: Fig. S1B) without affecting cell viability (Additional file 4: Fig. S1C). TOVC cells express much lower levels of DDR2 compared with OVCA433 cells (Additional file 4: Fig. S1A), and WRG-28 treatment did not have any detectable cytotoxic effect (Additional file 4: Fig. S1D). To assess the antitumour activity of TOVC cells, we evaluated the production of IFN-γ, a cytokine secreted by activated T cells within the TME, which exerts antitumour effects by inhibiting tumour cell proliferation and angiogenesis, and by inducing apoptosis in cancer cells [33]. All TOVC cell lines (n = 6) specifically recognized OVCA433 cells, as evidenced by high IFN-γ production (mean = 700 pg ml^-1^) in the culture supernatant after 24 hours of culture with OVCA433 on Col1 (Fig. 1D). Interestingly, treatment of OVCA433 cells with WRG-28 significantly (p < 0.0001) enhanced IFN-γ production (mean = 1139 pg ml^-1^) by TOVC cells (Fig. 1D). No IFN-γ secretion was observed when primary T cell precursors were cultured with OVCA433 before TOVC generation (T + OVCA433 Col1), strongly supporting the tumour specificity of the TOVC cell lines (Fig. 1D).

**Fig. 2:**
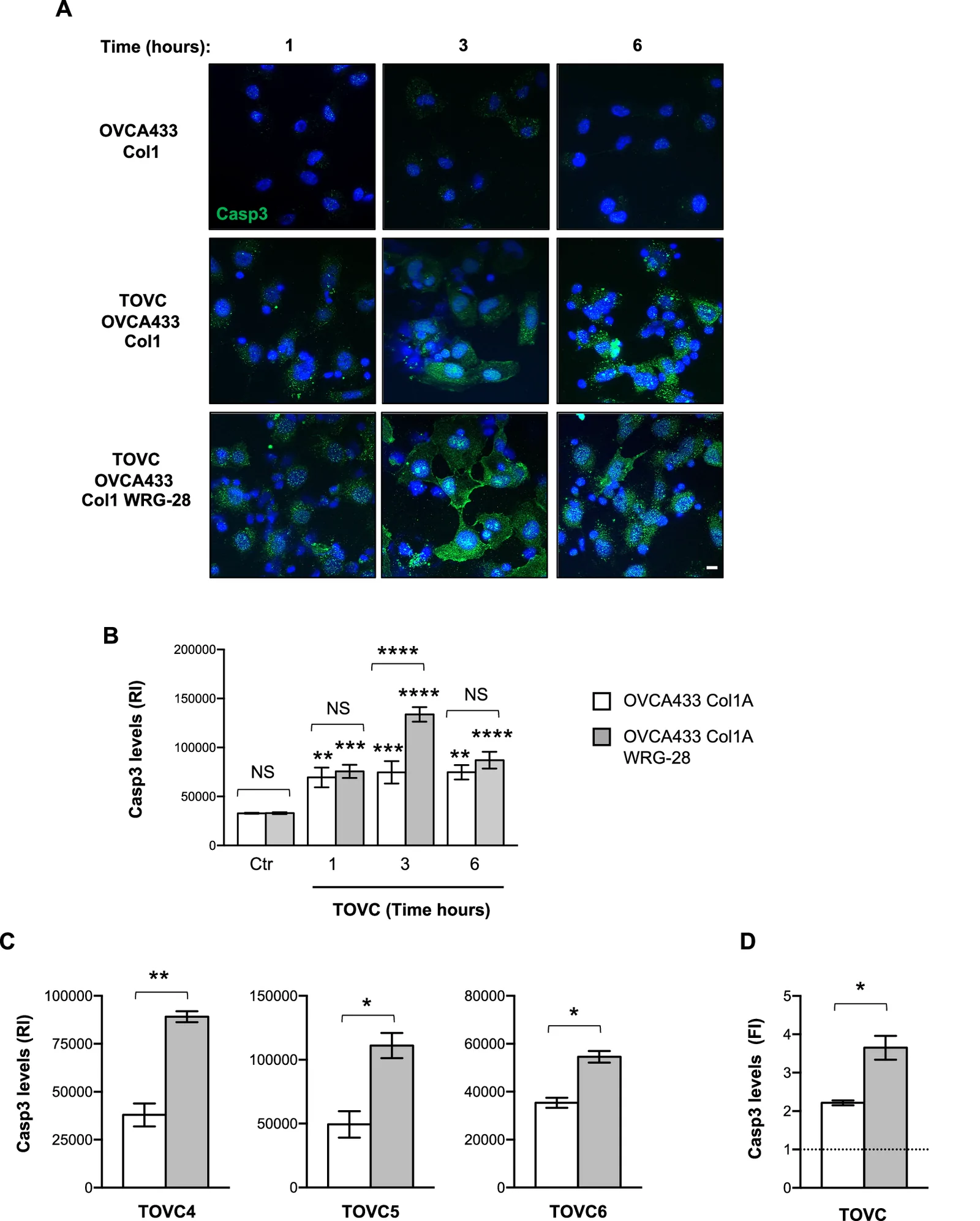
Inhibition of DDR2-Col1 interaction by WRG-28 enhances T-cell mediated apoptosis of OVCA433. (**A-D**) Confocal microscopy analysis of active Caspase 3 (Casp3) levels in OVCA433 cells cultured on Col1-coated coverslips for the indicated times (**A, B**) or 3 hours (**C**) with or without the indicated TOVC cell lines in the presence of DMSO (vehicle control) or WRG-28. Nuclei were stained with DAPI (blue). Scale bar = 10 μm. Nuclear Casp3 levels relative intensity (RI) was quantified and averaged per microscopic field. Data are presented as mean RI ± SEM from at least four microscopic fields. (**D**) Fold inductions (FI) of Casp3 levels in OVCA433 on Col1 co-cultured with three independent TOVC cell lines, relative to OVCA433 cells growth on Col1 alone. Data are presented as mean ± SEM from three independent experiments. Statistical significance was determined by one-way ANOVA (**B**), or Student’s t test (**C-D**). * *p* < 0.05, *** *p* < 0.001, **** *p* < 0.0001. NS = not significant

We next evaluated the effect of DDR2/Col1 inhibition on the cytotoxic capacity of TOVC cell lines against OVCA433 cells. To this end, TOVC cells were co-cultured for the indicated times with OVCA433 cells adhered overnight to Col1-coated coverslips, in the presence or absence of WRG-28. Active caspase-3 (Casp3) levels in OVCA433 cells were then assessed by fluorescence microscopy (Fig. 2A). Kinetic analysis revealed increased Casp3 activation in OVCA433 cells following co-culture with TOVC cells (Fig. 2A–C). WRG-28 treatment significantly enhanced Casp3 levels ( ~ 1.6-fold; p < 0.05) in OVCA433 cells after 3 hours of co-culture with all analysed TOVC cell lines (Fig. 2C, D). Notably, the enhanced Casp3 levels observed following WRG-28 treatment decreased at the 6-hour time point, potentially reflecting an earlier and transient induction of apoptotic signalling following DDR2 inhibition.

These findings demonstrate that the inhibition of DDR2/Col1 interaction in OVCA433 cells enhances the activation and cytotoxic function of tumour-specific T cells.

### Inhibition of DDR2/Col1 interaction sensitises SOC to TOVC-mediated cytotoxicity

Our data on the increase of Casp3 levels in OVCA433 on Col1 cultured with TOVC cells in the presence of WRG-28 (Fig. 2), suggested that inhibition of DDR2/Col1 interaction could enhance the recruitment and cytotoxic activity of tumour-specific T cells. To verify this, we generated OVCA433-specific TOVC cell lines as described above (Fig. 1A), and following two rounds of stimulation, CD8-enriched TOVC cells were obtained by depleting CD4^+^ T cells by immunomagnetic sorting. Compared with primary CD8^+^ T cell precursors (T0), CD8-enriched TOVC cells (n = 6) were 74% pure as shown by anti-CD3/CD8 staining and expressed CD28 (80%) on the cell surface (Additional file 4: Fig. S2A-B).

**Fig. 3:**
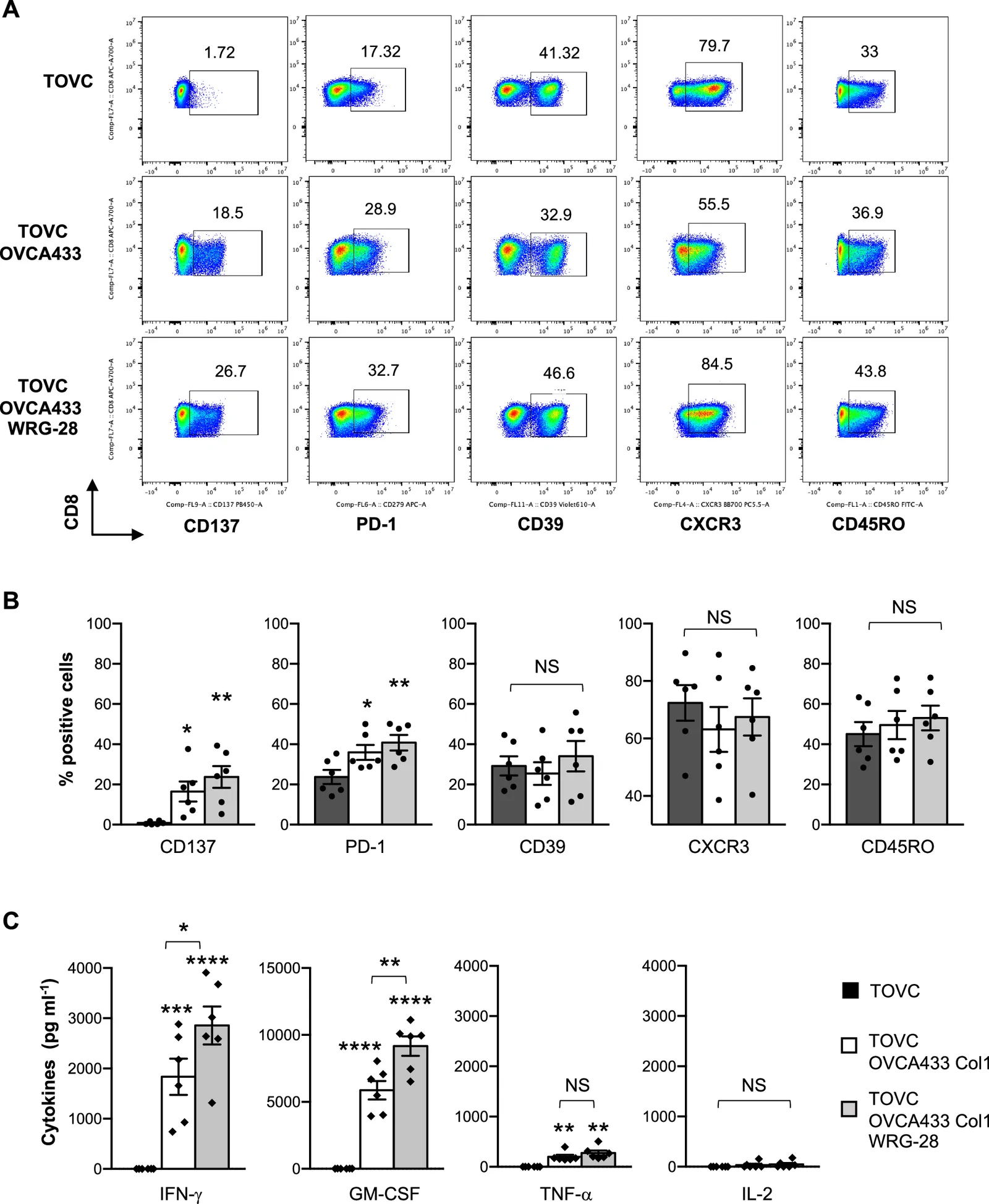
WRG-28 enhances OVCA433-specific T-cell responses. (**A**) Representative flow cytometry profiles of the indicated markers in donor-derived T cell precursors prior to TOVC generation (T0) and CD8-enriched TOVC cells after two rounds of stimulation with OVCA433 cells. Numbers within dot plots indicate the percentage of positive cells. (**B**) Quantification of the proportion of cells expressing the indicated markers in six independent CD8-enriched TOVC cell lines. (**C**) Cytokine production (IFN-γ, GM-CSF, TNF-α, IL-2) in culture supernatants of CD8-enriched TOVC cell lines (n = 6) stimulated for 24 h with OVCA433 on Col1 in presence of DMSO (vehicle control) or WRG-28. Data are presented as mean ± SEM from six independent experiments. Statistical significance was determined by one-way ANOVA. * *p* < 0.05, ** *p* < 0.01, *** *p* < 0.001, **** *p* < 0.0001. NS = not significant

Additionally, analysis of surface activation markers associated with tumour-specific CD8^+^ T cells [30,34,35] revealed a significant upregulation of CD45RO (mean %: T0 = 24.93 ; TOVC = 45.35), CXCR3 (mean %: T0 = 31.45; TOVC = 72.53), PD-1 (mean %: T0 = 17.43; TOVC = 28.68), CD137 (mean %: T0 = 0.32; TOVC = 1.03) and CD39 (mean %: T0 = 1.48; TOVC = 29.67) (Additional file 4: Fig. S2A-B). WRG-28 treatment significantly increased the percentage of CD137^+^ cells (mean %: TOVC = 0.9; TOVC OVCA433 Col1 = 16.44; TOVC OVCA433 Col1 WRG-28 = 23.71) and PD-1^+^ cells (mean %: TOVC = 23.68; TOVC OVCA433 Col1 = 35.92; TOVC OVCA433 Col1 WRG-28 = 40.75) in CD8-enriched TOVC cells stimulated with OVCA433 on Col1 (Fig. 3A, B). No significant changes in the proportion of CD45RO^+^, CXCR3^+^, or CD39^+^ cells were observed following stimulation. Moreover, DDR2 targeting significantly enhanced IFN-γ (mean pg ml^-1^: TOVC = 0.83; TOVC OVCA433 Col1 = 1835; TOVC OVCA433 Col1 WRG-28 = 2858) and GM-CSF production (mean pg ml^-1^: TOVC = 11.54; TOVC OVCA433 Col1 = 5871; TOVC OVCA433 Col1 WRG-28 = 9179) by all CD8-enriched TOVC cell lines (Fig. 3C). Conversely, TNF-α production was low (mean pg ml^-1^ = 214) and was not significantly altered by WRG-28 treatment, while IL-2 production was undetectable under all conditions (Fig. 3C).

**Fig. 4:**
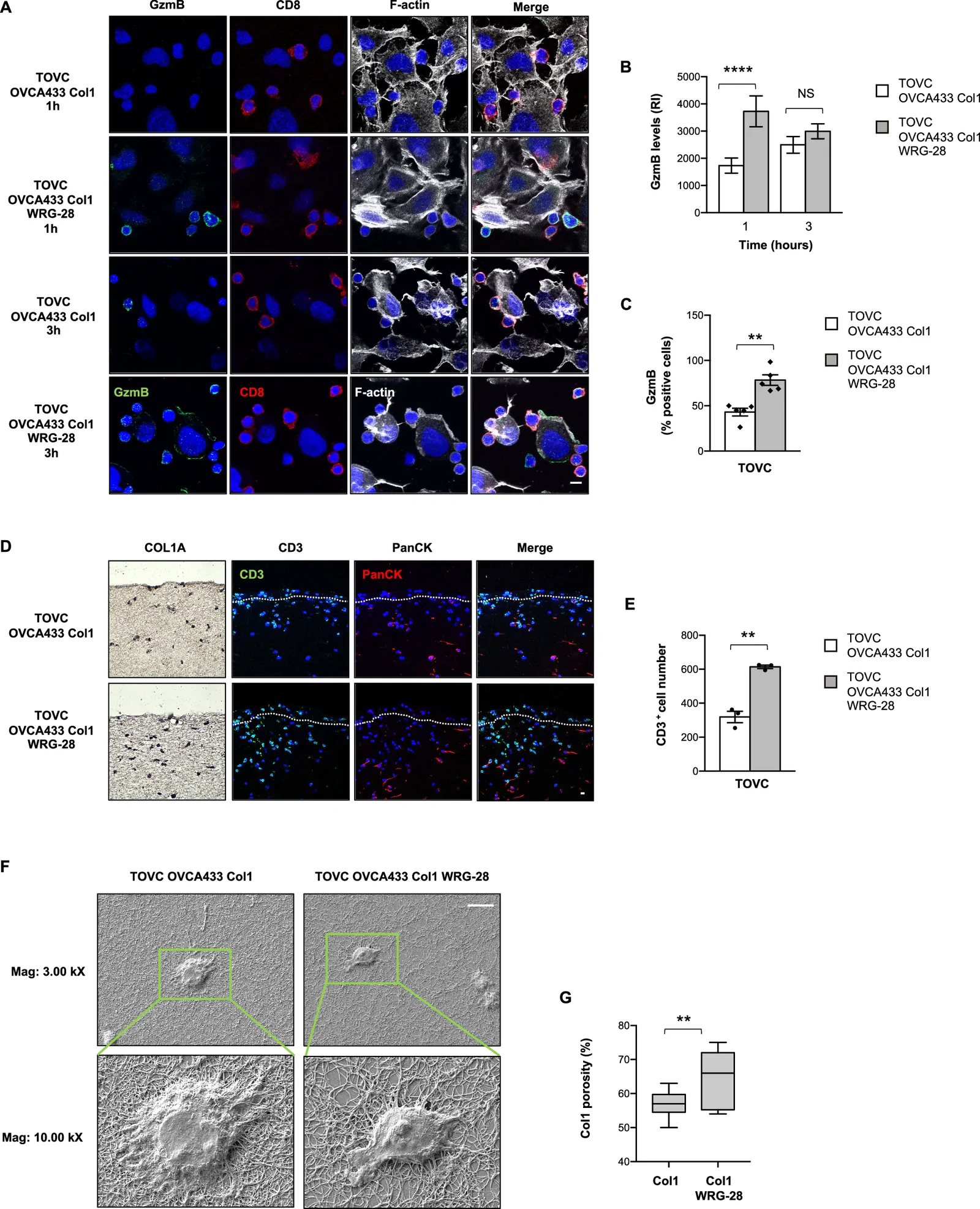
WRG-28 potentiates the recruitment and cytotoxic activity of OVCA433-specific CD8-enriched TOVC cells. (**A-C**) Confocal microscopy analysis of GzmB (green) and F-actin (white) in CD8^+^ TOVC cells (red) stimulated with OVCA433 cells on Col1 in the presence of DMSO (vehicle control) or WRG-28 for the indicated times (**A, B**) or for 1 h (**C**). Nuclei were stained with DAPI (blue). Scale bar = 10 μm. (**B**) Quantification of GzmB relative intensity (RI) in CD8^+^ TOVC cells. RI values were averaged per microscopic field. Data represent mean RI ± SEM from at least five microscopy fields per condition. Statistical significance was determined by one-way ANOVA. (**C**) Proportion of GzmB-positive CD8^+^ cells in five independent TOVC cell lines (n = 5). Data are presented as mean ± SEM from five independent experiments. Statistical significance was determined by Student’s t test. (**D, E**) Three independent CD8-enriched TOVC cells were added above OVCA433-populated hydrogels and cultured for 8 h in the presence of DMSO (vehicle control) or WRG-28. Representative immunostaining of Col1, CD3 (green) and pan-cytokeratin (PanCK; red) is shown. Nuclei were stained with DAPI (blue). Scale bar = 10 μm. (**E**) Quantification of infiltrating CD3^+^ TOVC cells within OVCA433-populated hydrogel sections. Data are presented as mean ± SEM from three independent experiments. Statistical significance was determined by Student’s t test. (**F**) Representative scanning electron microscopy images of hydrogel sections acquired at 10.00 and 3.00 kX magnifications (Mag). Scale bar = 30 μm. (**G**) Hydrogel porosity quantified from 12 scanning electron microscopy micrographs obtained from three independent experiments. Data are presented as median values, and statistical significance was determined by Mann-Whitney test. * *p* < 0.05, ** *p* < 0.01, **** *p* < 0.0001

We next evaluated the cytotoxic activity of OVCA433-specific CD8-enriched TOVC cells by quantifying granzyme B (GzmB), a serine protease released by cytotoxic T cells activating caspase-3–dependent apoptosis [36]. Kinetic analysis revealed that GzmB levels peaked after 1 hour in CD8-enriched TOVC cells co-cultured with OVCA433 on Col1 (Fig. 4A, B) and paralleled with F-actin reorganisation at the TOVC–OVCA433 interface (Fig. 4A). WRG-28 treatment significantly enhanced GzmB levels across all CD8-enriched TOVC (n = 5), resulting in approximately a two-fold increase in GzmB⁺ cells (mean %: TOVC OVCA433 Col1 = 39.28; TOVC OVCA433 Col1 + WRG-28 = 74.43) (Fig. 4C).

To determine whether this enhanced cytotoxicity was linked to changes in the collagen matrix [37], we examined TOVC cell recruitment into OVCA433-populated hydrogels. Immunohistochemical analysis revealed reduced Col1 staining in WRG-28-treated hydrogels, accompanied by a marked increase in CD3⁺ TOVC cell infiltration, with an approximately two-fold rise in recruited cells compared with controls (mean: TOVC OVCA433 Col1 = 324.7; TOVC OVCA433 Col1 + WRG-28 = 649.3) (Fig. 4D, E). Scanning electron microscopy analysis showed a reduction in collagen fibre density surrounding OVCA433 cells following WRG-28 treatment (Fig. 4F). Quantitative structural analysis of the collagen hydrogel architecture based on scanning electron microscopy micrographs demonstrated a significant increase in scaffold porosity in WRG-28-treated hydrogels (Fig. 4G), indicating a less compact collagen matrix architecture. Notably, RNA-seq analysis for the differentially expressed genes (DEGs) in OVCA433 cells grown on Col1 in the presence of WRG-28 compared to those grown on Col1 alone showed a significant (FDR < 0.05 and FC > 1.5) downregulation of 101 genes and upregulation of 46 genes (Additional file 2: Table 2). Among the downregulated genes, we identified a cluster of DEGs associated with ECM organization (GO:0030198; GO:0030213) and remodelling (GO:0022617; GO:0030574), such as MMP9, ADAMTS15, SPARC, TNFAIP6, and HAS2 [38–42]. Gene Ontology enrichment analysis further identified ECM-associated cellular components among the significantly downregulated pathways, supporting a potential role of DDR2 in ECM remodelling (Additional file 4: Fig. S3).

These findings demonstrate that inhibition of DDR2/Col1 interaction by WRG-28 enhances both the recruitment and cytotoxic activity of tumour-specific CD8⁺ T cells, alongside changes in collagen matrix architecture and ECM-associated gene expression.

### Immunotherapeutic efficacy of combined WRG-28 and CD28xMUC1 BsAbs

**Fig. 5:**
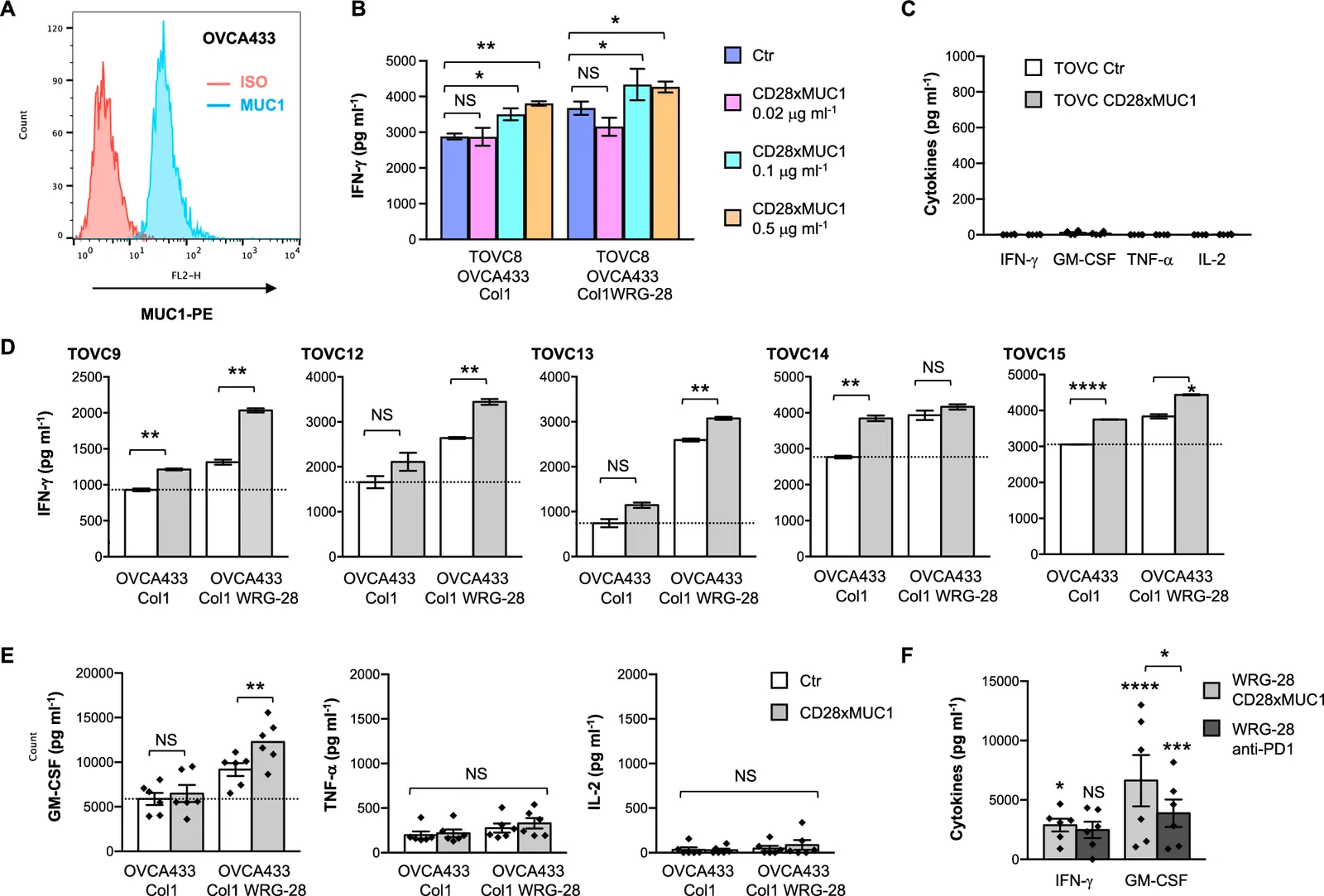
DDR2 inhibition enhances OVCA433-specific T cell response to CD28xMUC1 BsAbs. (**A**) Flow cytometry analysis of MUC1 surface expression in OVCA433 cells. Isotype control Ab staining (ISO-PE) is shown. (**B**) IFN-γ production by a representative CD8-enriched TOVC cell line stimulated for 24 h with OVCA433 on Col1 and 500 ng ml^-1^ isotype control Fab2 Ig (Ctr), WRG-28, or the indicated concentrations of CD28xMUC1 BsAbs, alone or in combination with WRG-28. Data are presented as mean ± SEM from triplicate cultures. (**C-E**) Cytokine production (IFN-γ, GM-CSF, TNF-α, IL-2) by five independent CD8-enriched TOVC cells stimulated for 24 h with 500 ng ml^-1^ isotype control Fab2 Ig (Ctr), CD28xMUC1 BsAbs alone (**C**), or in co-culture with OVCA433 on Col1 (**D, E**). Data are presented as mean ± SEM from five independent experiments performed in triplicate. (**D**). (**F**) IFN-γ and GM-CSF production by CD8-enriched TOVC cells (n = 6) stimulated for 24 h with OVCA433 on Col1 in the presence of WRG-28 combined with CD28xMUC1 BsAbs or 10 μg ml-1 pembrolizumab (anti-PD1). Data are presented as mean ± SEM from six independent experiments performed in triplicate. Statistical significance was determined by one-way ANOVA. * *p* < 0.05, ** *p* < 0.01, *** *p* < 0.001, **** *p* < 0.0001. NS = Not significant

DDR2 inhibition has been shown to enhance the efficacy of PD-1-targeted immunotherapy in preclinical models of breast, melanoma and colon cancer [14]. We next evaluated whether DDR2 blockade could sensitise SOC to immunotherapy. Given the poor response rates of ovarian cancer to PD-1 or PD-L1 blockade [15], we adopted an immunotherapeutic approach based on CD28xTAA bispecific antibodies (BsAbs) [17]. Specifically, we used a recombinant tandem Fab2 BsAb targeting CD28 and mucin 1 (MUC1), a TAA that promotes progression and metastasis in ovarian cancers [43], and is highly expressed on the surface of OVCA433 cell line (Fig. 5A).

Dose-response analysis showed that CD28xMUC1 BsAbs significantly increased IFN-γ production by TOVC cells cultured with OVCA433 on Col1 at concentrations of 0.1 and 0.5 μg ml^-1^ (Fig. 5B). In contrast to agonistic or superagonistic Abs, which induce high levels of inflammatory cytokines in human [44], CD28xMUC1 BsAbs alone did not elicit detectable production of IFN-γ, GM-CSF, TNF-α or IL-2 in TOVC cells (Fig. 5C). Notably, CD28xMUC1 BsAbs enhanced OVCA433-specific T cell responses, particularly when combined with WRG-28, as demonstrated by significant increases in IFN-γ (Fig. 5B, D) and GM-CSF production (Fig. 5E). In contrast, no significant changes were observed in TNF-α or IL-2 levels across treatment conditions (Fig. 5E). Importantly, the combination of CD28×MUC1 BsAbs and WRG-28 induced higher levels of IFN-γ and GM-CSF production than the combination of anti-PD-1 and WRG-28 (Fig. 5F), indicating a stronger cytokine response in this experimental system.

**Fig. 6:**
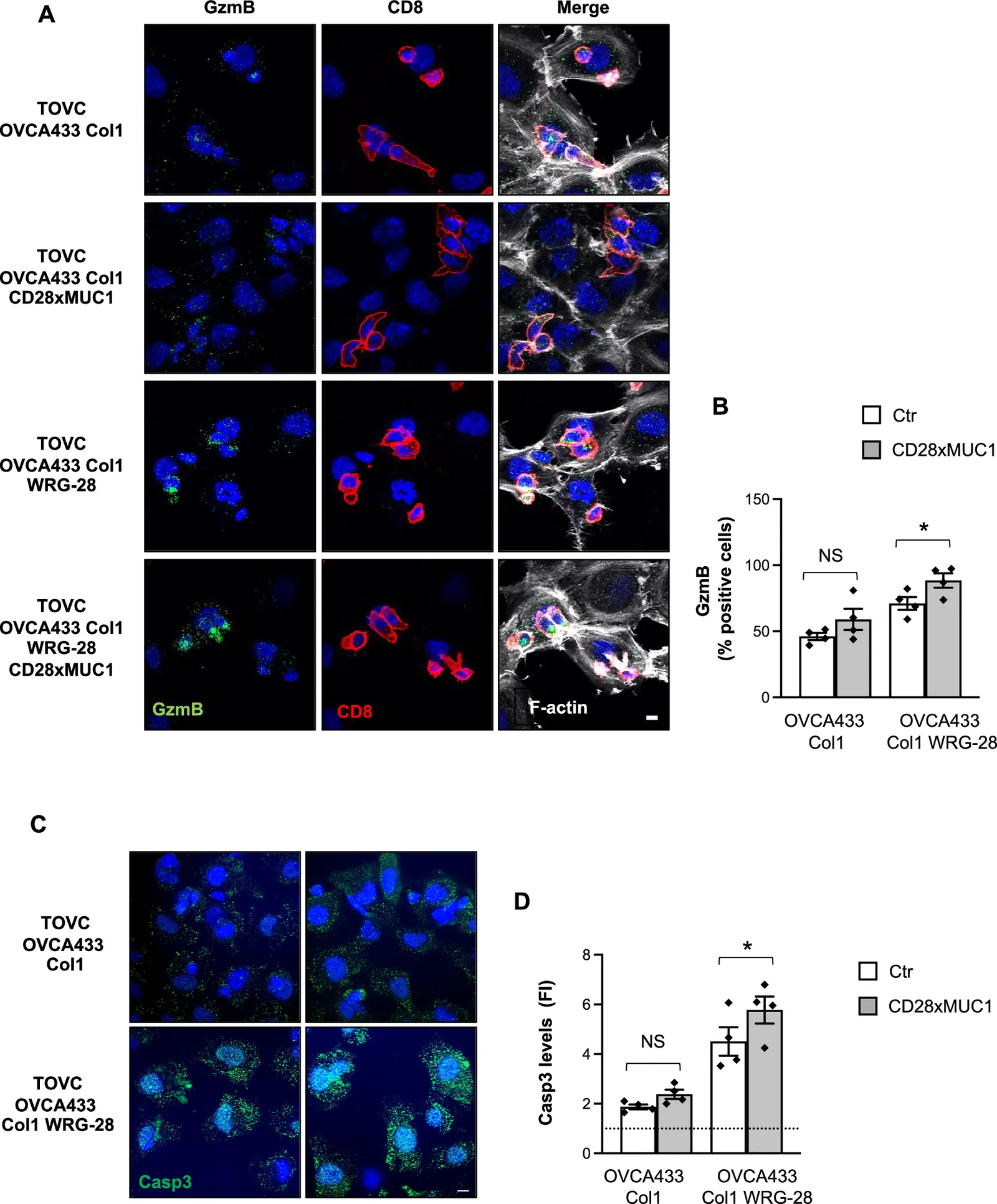
WRG-28 in combination with CD28xMUC1 BsAbs enhances the cytotoxic activity of OVCA433-specific CD8-enriched TOVC cells. (**A-D**) Confocal microscopy analysis of GzmB (green), CD8 (red) and F-actin (white) (**A, B**), or Casp3 (green) (**C, D**) in four independent CD8-enriched TOVC cell lines co-cultured for 1 h (**A, B**) or 3 h (**C, D**) with OVC433 cells on Col1-coated coverslips in the presence of isotype control Fab2 Ig (Ctr), WRG-28, CD28xMUC1 BsAbs, or the combination treatment. Nuclei were stained with DAPI (blue). Scale bar = 10 μm. (**B**) Quantification of the proportion of GzmB-positive CD8+ TOVC cells. (**D**) Fold inductions (FI) of Casp3 levels relative to control conditions. Data are presented as mean ± SEM from four independent experiments. Statistical significance was determined by one-way ANOVA. * *p* < 0.05. NS = not significant

Moreover, WRG-28-mediated DDR2 blockade in combination with CD28xMUC1 BsAb significantly enhanced either GzmB release (Fig. 6A-B), and Casp3 levels in OVCA433 cells cultured with TOVC cells on Col1 (Fig. 6C-D), supporting enhanced tumour cell death under conditions of collagen-mediated immune restriction.

### Efficacy of combined CD28xMUC1 BsAbs and DDR2 inhibition in HG-SOC-specific T cell responses

High-grade serous ovarian carcinoma (HG-SOC) accounts for 60–80% of epithelial ovarian cancers and, compared with LG-SOC, is associated with higher recurrence rates and rapid intraperitoneal dissemination [2]. To evaluate the immunotherapeutic potential of dual targeting in this setting, we assessed the efficacy of WRG-28 and CD28xMUC1 BsAbs in an HG-SOC model. The HLA-A2^+^ OVCAR3 cell line, which recapitulates the major genomic features of HG-SOC [28], expresses both MUC1 and DDR2 (Additional file 4: Fig. S4A-B), undergoes DDR2 tyrosine phosphorylation upon Col1 binding and is sensitive to WRG-28 treatment (Additional file 4: Fig. S4C-D) that does not exerts significant cytotoxic effects (Additional file 4: Fig. S4E). The transcriptomic profile of OVCAR3 cells treated with WRG-28 revealed a high degree of similarity to that observed in OVCA433 cells. Notably, WRG-28 treatment significantly downregulated genes involved in ECM organization and remodelling, including MMP1, MMP7, MMP10, SPARC, FAP, and COL14A1 [45,46] (Additional file 4: Fig. S5 and Additional file 3: Table 3), further supporting ECM remodelling following DDR2 inhibition.

**Fig. 7:**
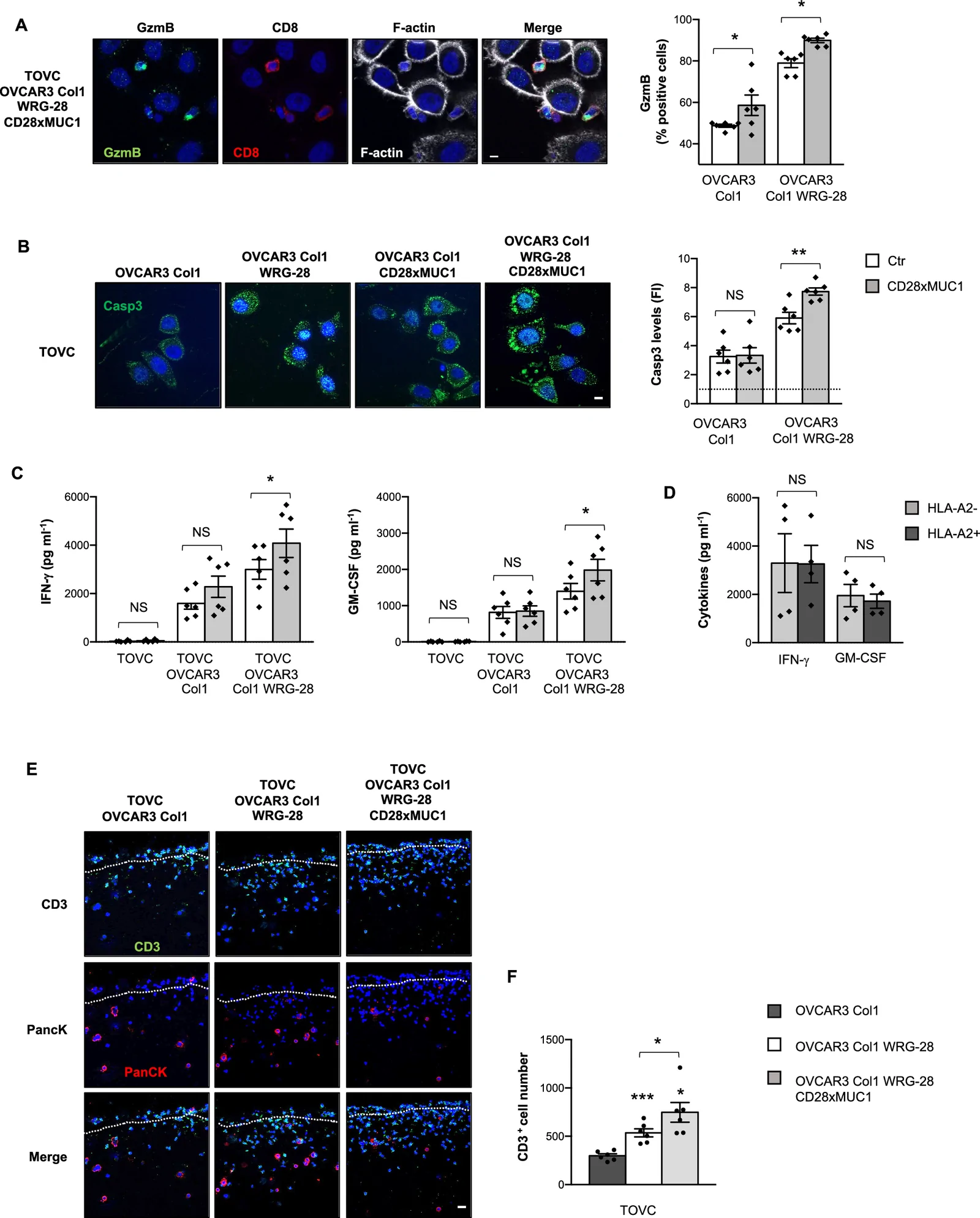
WRG-28 in combination with CD28xMUC1 BsAbs sensitises HG-SOC cells to tumour-specific T cell cytotoxicity. (**A, B**) Confocal microscopy analysis of GzmB (green) and F-actin (white) (**A**), or Casp3 (green) (**B**) in CD8-enriched TOVC cell lines generated from four HLA-A2-matched and two non-HLA-matched donors co-cultured for 1 h (**A**) or 3 h (**B**) with OVCAR3 cells on Col1-coated coverslips in the presence of isotype control Fab2 Ig (Ctr), WRG-28, CD28xMUC1 BsAbs alone, or the combination treatment. Quantification of the proportion of GzmB-positive cells (**A**) and fold induction (FI) of Casp3 levels (**B**) was performed. (**C**) IFN-γ and GM-CSF production by CD8-enriched TOVC cell lines (n = 6) co-cultured for 24 h with OVCAR3 on Col1 in the presence of isotype control Fab2 Ig (Ctr), WRG-28, CD28xMUC1 BsAbs alone, or the combination treatment. (**D**) Comparisons of IFN-γ and GM-CSF production by TOVC cell lines from HLA-A2-matched and non-HLA-matched donors following stimulation with OVCAR3 on Col1 in the presence of combined WRG-28 and CD28xMUC1 BsAbs. (**E, F**) Confocal microscopy analysis of CD3 (green) and PanCK (red) in OVCAR3-populated hydrogels co-cultured for 8 h with six independent TOVC cells in the presence of isotype control Fab2 Ig (Ctr), WRG-28, CD28xMUC1 BsAbs alone, or the combination treatment. Nuclei were stained with DAPI (blue). Scale bar = 10 μm. (**F**) Quantification of infiltrating CD3⁺ TOVC cells within hydrogel sections is shown. Data are presented as mean ± SEM from six independent experiments. Statistical significance was determined by one-way ANOVA. * *p* < 0.05, *** *p* < 0.001. NS = not significant

OVCAR3-specific CD8-enriched TOVC cells were generated by priming HD-derived PBMCs with MUC1-positive OVCAR3 cells, as previously described for LG-SOC-specific T cells (Fig. 1A). Among these, four CD8-enriched TOVC cell lines were generated from HLA-A2^+^ donor PBMCs matched to HLA-A2^+^ OVCAR3 cells. Consistent with results from LG-SOC-specific T cells, co-treatment with WRG-28 and CD28xMUC1 BsAbs significantly enhanced GzmB release (Fig. 7A), Casp3 activation (Fig. 7B), and cytokine production (Fig. 7C) following OVCAR3 stimulation. Importantly, the majority of TOVC cell lines used in cytotoxicity experiments (four out of six; Fig. 7A, B) were generated from HLA-A2^+^ PBMCs, and comparable cytokine production was observed between HLA-A2-matched and non-matched TOVC cell lines co-treated with WRG-28 and CD28xMUC1 BsAbs (Fig. 7D), suggesting that the functional responses observed were not predominantly driven by allorecognition.

The analysis of TOVC cell recruitment in OVCAR3-populated hydrogels confirmed the findings obtained from OVCA433, showing an enhanced CD3⁺ TOVC cell infiltration in the presence of WRG-28 that further increased with the combination of CD28xMUC1 BsAbs (Fig. 7E, F).

Together, these findings support the potential combining DDR2 inhibition with T-cell costimulation using CD28xMUC1 BsAbs to enhance the recruitment, cytotoxic activity, and effector functions of HG-SOC-specific T cells.

### DDR2 expression is associated with poor prognosis and an immune-excluded phenotype in SOC

**Fig. 8:**
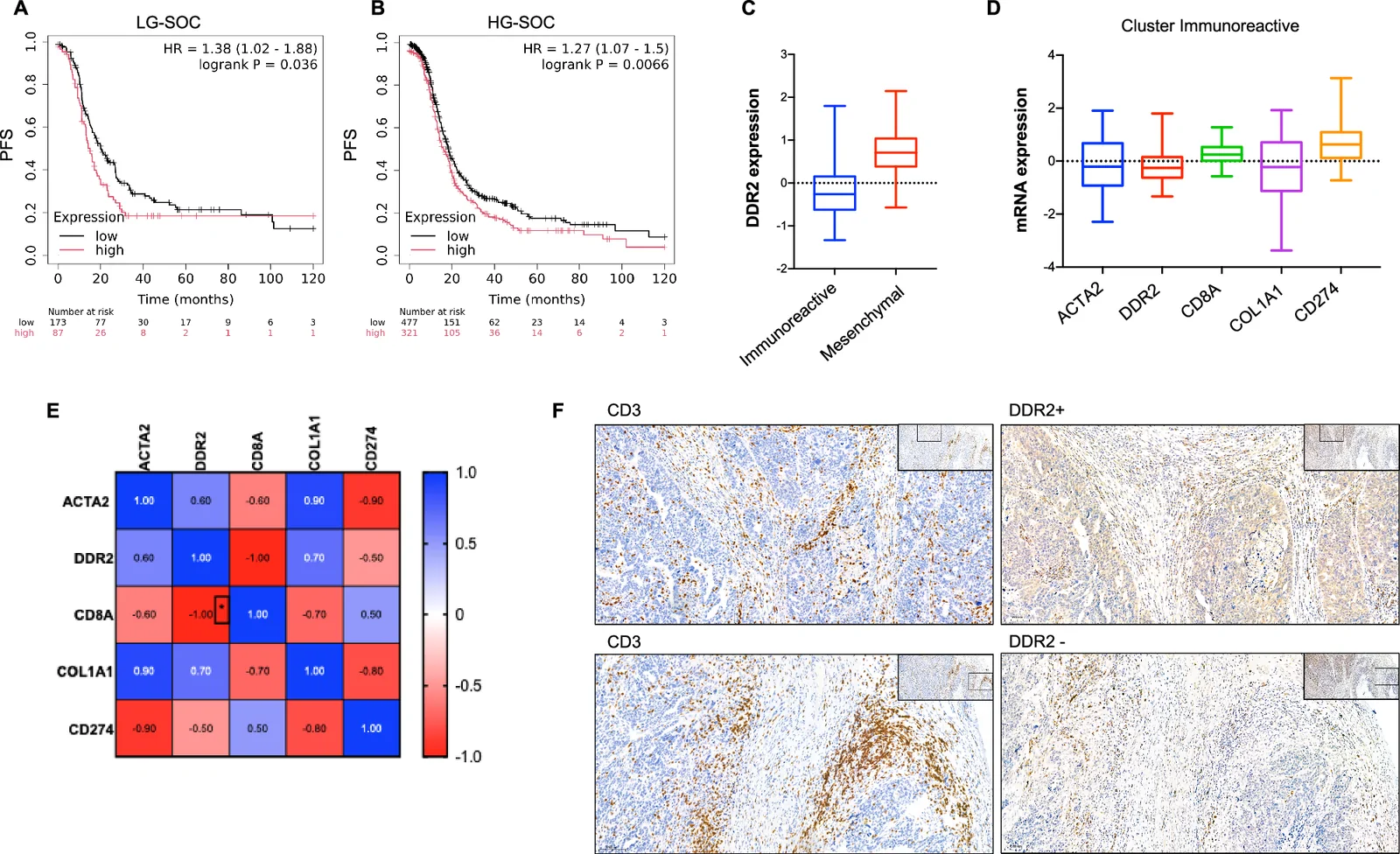
DDR2 expression is associated with poor prognosis and an immune-excluded phenotype in SOC. (**A, B**) Kaplan–Meier analysis of progression-free survival (PFS) according to DDR2 expression levels in LG-SOC (**A**) and HG-SOC (**B**). High DDR2 expression is associated with reduced PFS in both cohorts. Statistical significance was determined using the log-rank test. (**C**) DDR2 expression across molecular subtypes of SOC, showing significant enrichment in the mesenchymal subtype compared with the immunoreactive subtype. Statistical significance was determined using the Mann–Whitney test. (**D, E**) Correlation analysis between DDR2 and CD8A expression within the immunoreactive subgroup, demonstrating a significant inverse association. Statistical significance was determined using Spearman’s correlation test. (**F**) Representative immunohistochemical staining of primary HG-SOC tumour samples for DDR2 and CD3. Regions with high DDR2 expression in tumour cells showed reduced peri-tumoral CD3⁺ T-cell infiltration, whereas areas with low DDR2 expression exhibited increased CD3⁺ cell accumulation, supporting an inverse association between DDR2 expression and T-cell infiltration

To assess the clinical relevance of DDR2 expression in SOC, we analysed the association between DDR2 expression levels and clinical outcomes using Kaplan–Meier survival analysis and log-rank tests in publicly available ovarian cancer datasets (www.kmplot.com). Patients were stratified according to low or high DDR2 expression, and progression-free survival (PFS) was evaluated in both LG- and HG-SOC cohorts. High DDR2 expression was associated with poorer clinical outcomes in both LG- and HG-SOC (Fig. 8A, B). In LG-SOC, elevated DDR2 expression correlated with reduced PFS (HR = 1.38, p = 0.036), whereas a similar but more significant association was observed in HG-SOC (HR = 1.27, p = 0.0066). In both cohorts, patients with high DDR2 expression exhibited a more rapid decline in progression-free probability, supporting an association between DDR2 expression and adverse clinical outcomes. We also investigated the clinical impact of combined DDR2 and MUC1 expression on PFS (Additional file 4: Fig. S7). In LG-SOC, high DDR2/MUC1 co-expression showed a trend towards poorer PFS (HR = 1.37), although this did not reach statistical significance (p = 0.1). In contrast, in HG-SOC, elevated DDR2/MUC1 expression was associated with improved PFS (HR = 0.66, p = 0.0073), suggesting subtype-specific associations that warrant further investigation. However, further studies in independent and prospectively collected cohorts will be required to determine the biological and clinical significance of DDR2/MUC1 co-expression and to assess its potential utility as a biomarker for patient stratification or predicting therapeutic response.

Stratification by molecular subtype of SOC revealed that DDR2 expression was significantly enriched in the mesenchymal cluster compared with the immunoreactive subtype (Fig. 8C). These data are consistent with the established role of DDR2 in collagen signalling and ECM remodelling. Within the immunoreactive subgroup, DDR2 expression inversely correlated with immune-related markers, including CD8A (Fig. 8D, E), supporting an association between DDR2 expression and reduced T-cell infiltration. In addition, correlations with CD274 (PD-L1) expression suggest a potential relationship between DDR2 signalling and immunosuppressive pathways.

To provide preliminary histological validation, DDR2 and CD3 expression were evaluated by immunohistochemistry in five primary HG-SOC samples. Regions with high DDR2 expression in tumour cells were associated with reduced peri-tumoral CD3⁺ T-cell infiltration, whereas areas with low or absent DDR2 expression exhibited increased CD3⁺ cell accumulation (Fig. 8F). These preliminary observations are consistent with an inverse association between DDR2 expression and T-cell infiltration in HG-SOC. However, the small sample size (n = 5) warrants confirmation in larger patients’ cohorts.

## Discussion

SOC is characterised by high recurrence rates and rapid intraperitoneal dissemination. Surgery is effective only for locoregional control, while chemotherapy has shown limited efficacy against metastatic disease, even when administered at low doses or with dose intensity limitations. These regimens can be harmful due to their inability to selectively target cancer cells while sparing normal tissues [47]. To reduce the severe adverse and cytotoxic effects of conventional chemotherapeutic agents, considerable efforts have been made to develop immunotherapeutic strategies that enhance immune responses to selectively inhibit tumour growth and/or eradicate malignant cells [48].

Ovarian cancers, including LG-SOC and HG-SOC, exhibit an immunoreactive phenotype [4,15] associated with the production of inflammatory chemokines such as CXCL9, CXCL10 and CXCL11. These chemokines mediate the recruitment of effector tumour-specific T cells through the CXCR3 receptor [27]. In addition, CD8⁺ TILs within the ovarian cancer microenvironment express activation markers, including CD137, CD39 and PD-1, together with effector molecules such as IFN-γ and perforin/GzmB [48–50]. Consistent with these observations, our tumour-specific CD8^+^ TOVC cell lines upregulated CXCR3, CD137, CD39 and PD-1 following priming with OVCA433. Furthermore, TOVC cell lines exhibited a cytotoxic profile, demonstrated by high IFN-γ production and GzmB release. In agreement with studies linking CD137 expression to tumour reactivity, patient survival, and improved responses to immunotherapy in solid tumours [49–51], CD137 expression was significantly upregulated in CD8^+^ TOVC cell lines after OVCA433 stimulation. While the specific tumour antigens recognised by TOVC cell lines remain unidentified, owing to the heterogeneity of T cell receptors and potential neoantigens expressed in human ovarian cancers [52], we were able to reproducibly generate tumour-reactive T cells, hereby establishing a valuable *in vitro* model for testing immunotherapeutic approaches. Nevertheless, although this system enabled the generation of reproducible antigen-reactive T-cell populations, it may not fully recapitulate the phenotypic heterogeneity, dysfunction, and exhaustion state characteristic of ovarian cancer-associated T cells *in vivo*, which are shaped by the profoundly immunosuppressive TME [53–55]. Therefore, future studies employing patient-derived TILs, ascites samples, and primary tumour models will be important to validate the translational relevance of our findings.

In recent years, although immune checkpoint blockade has significantly improved patient prognosis in some solid tumours, the remission rate of PD-1/PD-L1 monotherapy is generally low, particularly in HG-SOC [56]. The ‘immune low-response state’ is evident in immune-excluded tumours, in which T cells are trapped in the stromal regions, hindering their entry into the tumour core. Effective T cell infiltration is essential for the success of immunotherapy. However, SOC may modulate the tumour stroma to reduce its permeability to TILs, in part through crosstalk with CAFs [57]. Increased ECM stiffness due to dense and crosslinked Col1 has been shown to impair the migration and infiltration of cytotoxic T cells [26,58]. DDR2 is involved in the remodelling of collagen fibres [37], and its silencing has been shown to reduce collagen deposition and enhance CD8^+^ T cell infiltration in breast cancer [39]. Interestingly, we found that the inhibition of DDR2 by WRG-28 reduces collagen density in SOC cultures and downregulates the expression of genes associated with ECM organization and remodelling. Furthermore, strategies to inhibit collagen crosslinking or target DDR2 have proven effective in enhancing T cell migration and anti-PD-1 therapy in various solid tumours [14,59]. We extend these findings to ovarian cancer by demonstrating that *in vitro* targeting of the DDR2/Col1 interaction potentiates immune responses. DDR2 is expressed in ovarian cancers, and its expression and activation have been associated with tumour cell proliferation, progression, and invasion [60,61]. However, the role of DDR2 in SOC-specific immune responses remains unclear. Here, we show that WRG-28, a selective inhibitor of DDR2/Col1 interaction, enhances the recruitment, activation and cytotoxicity of TOVC cells specific for both LG-SOC and HG-SOC. Analysis of public datasets further support a model in which DDR2 contributes to the establishment of a dense, collagen-rich and immuno-excluded tumour microenvironment, rather than a purely immune-desert phenotype, ultimately leading to poorer clinical outcomes. These clinical associations are consistent with the mechanistic model derived from the functional studies. Nevertheless, further mechanistic investigations will be necessary to validate this model and establish the extent to which DDR2 blockade enhances T-cell recruitment and cytotoxic activity and thereby contributes to patient outcomes.

Monoclonal antibodies (mAbs) targeting immune checkpoints such as CTLA-4, PD-1, and PD-L1 have revolutionised cancer treatment, significantly improving long-term survival with acceptable safety profiles [62]. However, in ovarian cancer, PD-1/PD-L1 blockade, either as monotherapy or in combination with chemotherapy, has shown limited efficacy in terms of progression-free and overall survival in multiple phase III clinical trials [63–66]. Furthermore, dual blockade with CTLA-4 and PD-1 has offered only marginal progression-free survival benefits and is associated with increased rates of grade 3 adverse events [67]. An alternative strategy to elicit tumour-specific immunity involves BsAbs that simultaneously target T cells and TAAs. Most BsAbs are designed to engage CD3 on T cells and a TAA on cancer cells, thereby activating T cells and redirecting them to tumours [62]. In ovarian cancer, CD3xTAA BsAbs have demonstrated efficacy *in vitro* and in animal models [68,69]. However, CD3-engaging BsAbs can trigger broad polyclonal T-cell activation independent of antigen specificity [70], and are frequently associated with high toxicity, including cytokine release syndrome and neurotoxicity [71].

A more selective approach employs BsAbs targeting a TAA and a costimulatory receptor, such as CD28 [17]. CD28-based costimulation may permit more selective enhancement of tumour-reactive T-cell responses by functioning in conjunction with antigen-specific T-cell receptor signalling [17]. These BsAbs may provide a novel means of re-engaging effector T cells with improved selectivity and reduced toxicity. Several CD28xTAA BsAb formats have been developed and tested in combination with CD3 BsAbs or anti-PD-1 therapies [17,18,72]. An EGFRxCD28 BsAb was shown to enhance the antitumour efficacy of mesothelin x CD3 BsAbs in multiple solid tumours, including ovarian cancer [73]. A MUC16xCD28 BsAb (REGN5668) is currently undergoing phase I–II clinical trials in ovarian cancer (ClinicalTrials.gov, NCT04590326; registered on 19 October 2020). In this context, our *in vitro* data demonstrate that the combination of CD28xMUC1 BsAbs and WRG-28 enhances the recruitment, activation and cytotoxicity of TOVC cells specific for both LG-SOC and HG-SOC. Data on TGCA suggest a grade-specific and potentially context-dependent role of DDR2/MUC1 co-expression in SOC. While elevated DDR2 expression alone is associated with poor prognosis and stromal-driven immune exclusion, the addition of MUC1 expression may reflect a tumour context more responsive to immune engagement or targeted immunotherapeutic approaches, particularly in HG-SOC, where immune modulation may play a greater role in disease control. Importantly, these data are consistent with the mechanistic framework of this study, in which MUC1 serves as the target of CD28×MUC1 bispecific antibodies. Thus, DDR2/MUC1 co-expression may identify tumours in which combined stromal remodelling through DDR2 inhibition and T-cell costimulation via CD28 engagement could be particularly beneficial. Although these observations require further validation in independent clinical cohorts, they support the rationale for investigating combined DDR2 blockade and MUC1-targeted immunotherapy in selected SOC patient subsets. Furthermore, safety considerations are particularly important for therapeutic strategies involving CD28 costimulation given the severe cytokine release syndrome observed following systemic administration of the CD28 superagonist TGN1412 in a phase I clinical trial [74]. However, the CD28xMUC1 BsAbs investigated here differ mechanistically from unrestricted systemic CD28, as T-cell costimulation is conditionally linked to tumour-associated MUC1 recognition. Supporting this notion, stimulation of TOVC cells with CD28xMUC1 BsAbs alone did not induce inflammatory cytokine production in our experimental system, suggesting limited nonspecific activation in the absence of productive tumour–immune cell engagement.

Although the present study focused on tumour-specific T-cell responses, other immune populations are likely to influence therapeutic responses to DDR2 blockade and CD28×MUC1 bispecific antibodies. Macrophages, dendritic cells, and NK cells are important regulators of antitumour immunity in ovarian cancer and are strongly influenced by extracellular matrix composition and stromal architecture [75,76]. It would be interesting to evaluate in future the effects of DDR2 inhibition on both stromal cells and innate immune cells.

## Conclusions

In summary, our findings reveal a preclinical mechanistic rationale for combining DDR2 inhibition with T-cell costimulation in both LG-SOC and HG-SOC. Pharmacological blockade of DDR2 not only enhanced the recruitment, activation, and cytotoxic activity of tumour-specific T cells but also synergised with CD28xMUC1 BsAbs to amplify antitumour responses. This dual-targeting approach supports the concept that targeting the extracellular matrix in combination with T-cell costimulation may represent a strategy to enhance the antitumour immune response in ovarian cancer. A limitation of our study is the absence of *in vivo* validation. Although our collagen-rich 3D and *ex vivo* models recapitulate key structural and immunological features of the SOC tumour microenvironment, they cannot fully capture the complexity of tumour–stroma–immune interactions occurring in vivo. Future studies using orthotopic and/or humanized ovarian cancer models will be necessary to determine whether DDR2 inhibition enhances T-cell infiltration and tumour control and whether it improves responses to CD28xMUC1 bispecific antibody therapy in vivo. In addition to further mechanistic investigations in preclinical models, clinical validation will be required to determine whether this mechanism operates in patients and to define its biological and therapeutic relevance.

## Supplementary information

Supplementary material 1: Additional file 1: Table 1. Information on antibodies used in multicolour flow cytometry.

Supplementary material 2: Additional file 2: Table 2. Transcriptomic profiling of OVCA433 following WRG-28 treatment.

Supplementary material 3: Additional file 3: Table 3. Transcriptomic profiling of OVCAR3 following WRG-28 treatment.

Supplementary material 4: Additional file 4: Figure S1-S7. Fig S1-DDR2 expression in OVCA433 and TOVC cells. Fig S2- Phenotypic characterization of CD8-enriched TOVC cell lines. Fig S3-Transcriptomic profiling of OVCA433 cells. Fig S4-Immunophenotype of OVCAR3 cells. Fig S5-Transcriptomic profiling of OVCAR3 cells. Fig S6-Original immunoblot data corresponding to Additional file 4: Fig.S4C. Fig S7-Prognostic impact of DDR2/MUC1 co-expression in SOC.

Supplementary material 5:Additional files: The online version contains additional files

## Data Availability

The datasets used and/or analysed during the current study are available from the corresponding author on reasonable request. The RNA-Seq accompanying this paper is available through NCBI’s Gene Expression Omnibus (GEO) repository, under accession number GSE328697 located at https://www.ncbi.nlm.nih.gov/geo/query/acc.cgi?acc=GSE328697.

## Abbreviations

SOC: Serous ovarian carcinoma
Col1: Collagen type I
TME: Tumour microenvironment
DDR2: Discoidin domain receptor 2
BsAbs: Bispecific antibodies
MUC1: Mucin 1
LG-SOC: Low-grade serous ovarian carcinoma
HG-SOC: High-grade serous ovarian carcinoma
CAFs: Cancer-associated fibroblasts
DDR1: Discoidin domain receptor 1
EMT: Epithelial-to-mesenchymal transition
TILs: Tumour-infiltrating lymphocytes
TAAs: Tumour-associated antigens
HD: Healthy donors
PBMCs: Peripheral blood mononuclear cells
Casp3: Active caspase 3
GzmB: Granzyme B
