# Supplementary material for: Boosting ovarian cancer immunotherapy via discoidin domain receptor 2 blockade and CD28 x mucin 1 bispecific antibodies
Source: BMC Med. 2026 Aug 24;24:598. doi: 10.1186/s12916-026-05183-2 (PMC13721643; doi:10.1186/s12916-026-05183-2)
Supplement: Supplementary file 1 [file 12916_2026_5183_MOESM4_ESM.pdf]

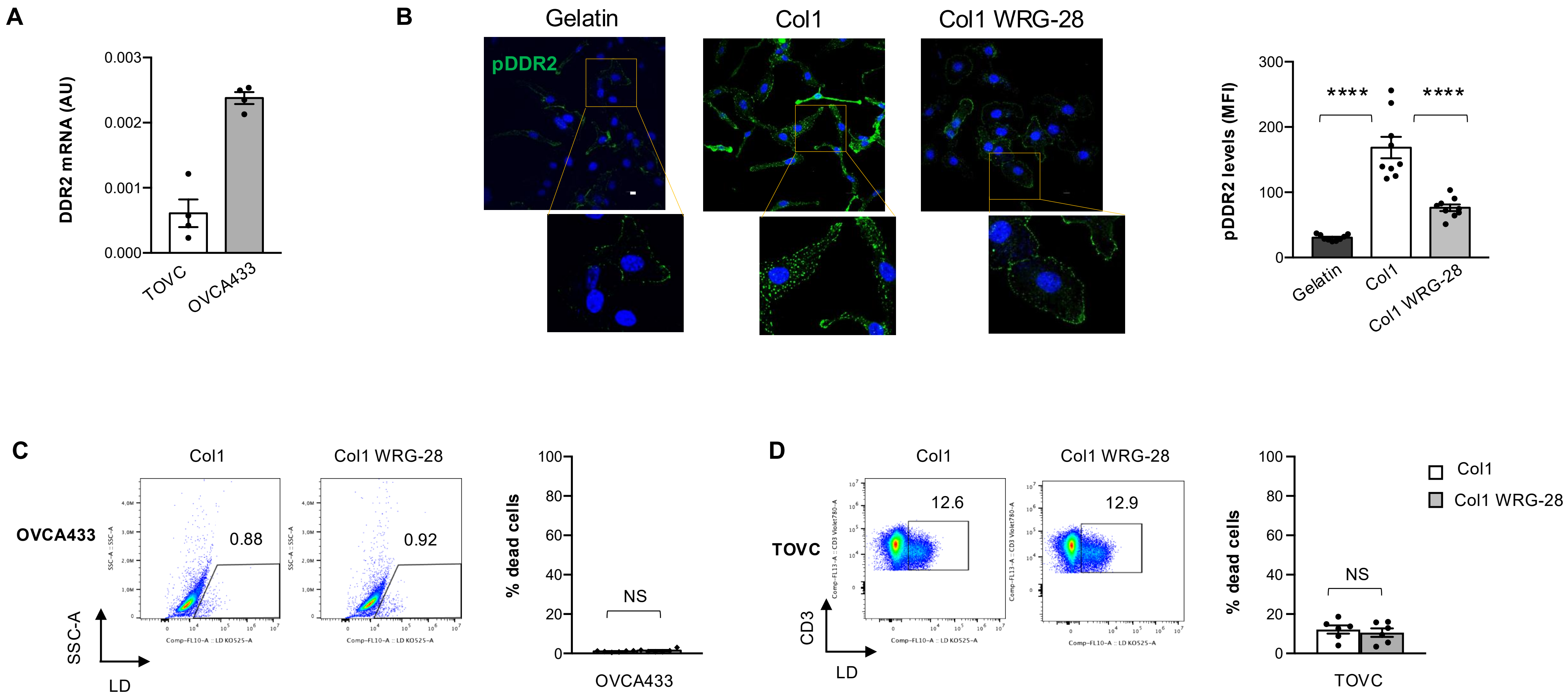

Fig. S1. DDR2 expression in OVCA433 and TOVC cells. (**A)** DDR2 mRNA levels in OVCA433 and TOVC cells were measured by real-time PCR and values, normalized to GAPDH, expressed as arbitrary units (AU). Data represent mean ± SEM (n = 4). (**B**) Representative immunofluorescence analysis of OVCA433 cells cultured on gelatin or Col1 (10 µg ml^{-1}) or Col1 with WRG-28 (2 µM) for 6 hrs and stained with anti-pDDR2 followed by Alexa Fluor 488 conjugated Abs (green). Nuclei were stained with DAPI (blue). Scale bar = 50 µm. Mean fluorescence intensity (MFI) of pDDR2 was quantified in at least 9 replicates and normalized to cell number. Data represent mean ± SEM (n = 9). Statistical significance was determined by one way ANOVA. (**C**, **D**) Representative flow cytometry analysis of OVCA433 cells (**C**) or TOVC cells (**D**) cultured for 24 hours on Col1 in the presence of DMSO (vehicle control) or WRG-28 (2 µM) and stained with Cyto3D LD (**C**) or anti-CD3 Brilliant Violet 787 and Fixable Aqua Live Dead (LD) kit (**D**) or. Data represent the mean percentage of dead cells ± SEM (n = 6). Statistical significance was determined by Student t test. ****$p < 0.0001$NS = not significant.

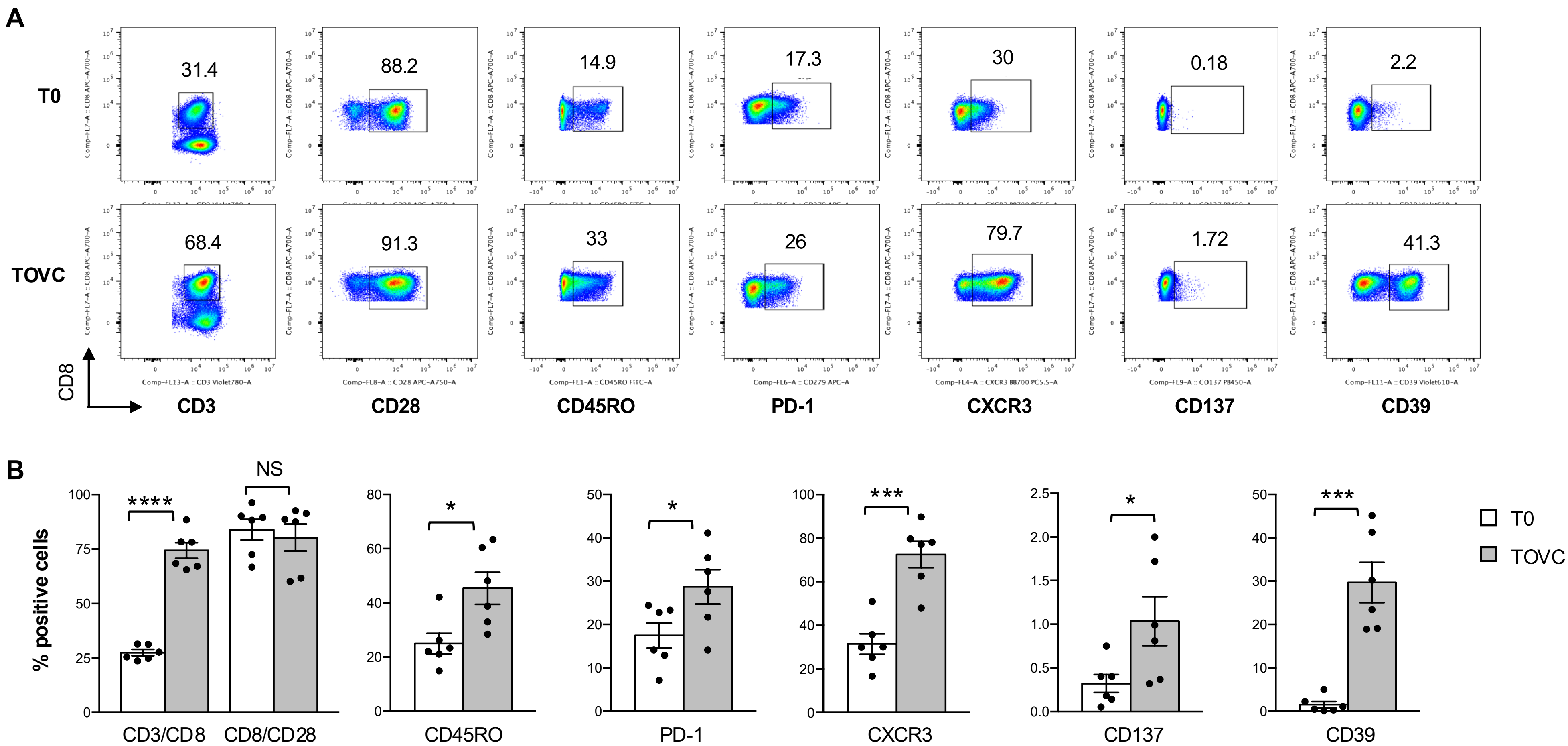

Fig. S2. Phenotypic characterization of CD8-enriched TOVC cell lines. (**A**) Representative multicolour flow cytometry of primary T cell precursors (T0) before stimulation and OVCA433-specific CD8-enriched TOVC cell lines after two rounds of stimulation with OVCA433 cells. Cells were stained with anti-CD3-BV786, anti-CD8-APC-Alexa700, anti-CD28-APC-Violet770, anti-CD45RO-FITC, anti-CXCR3-BB700, anti-CD279/PD-1-APC, anti-CD137-BV421, anti-CD39-BV605 Abs. Squares within the dot plots indicate the % of positive cells. (**B**) Data shown the mean % ± SEM (n = 5) and statistical significance was Student's t test. (*) $p < 0.05$, (***) $p < 0.001$, (****) $p < 0.0001$. NS = not significant

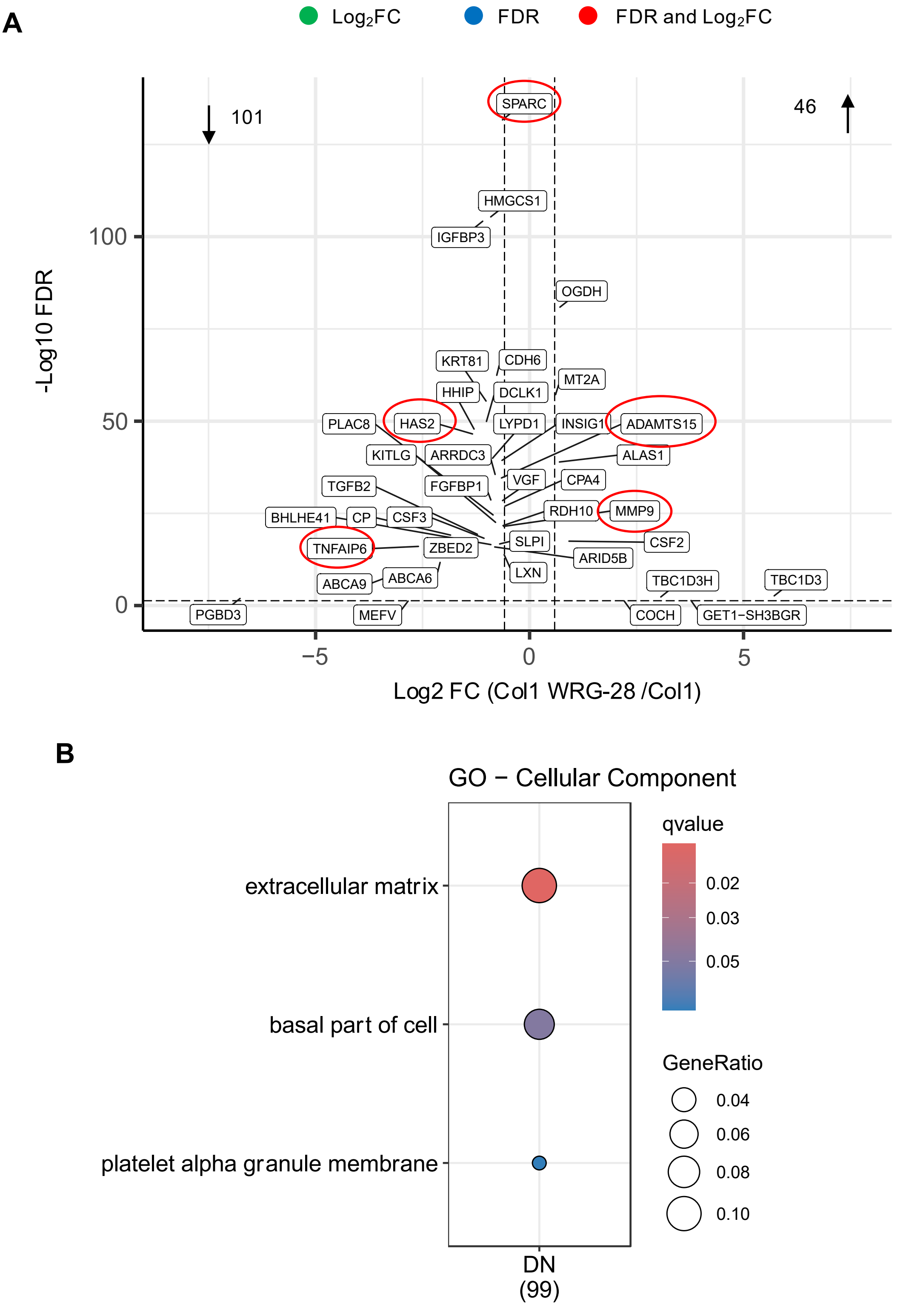

Fig. S3. Transcriptomic profiling of OVCA433 cells. (**A–B**) RNA-seq analysis of OVCA433 cells cultured on Col1 in the presence of DMSO (vehicle control) or 2 μM WRG-28 for 24 hours. (**A**) Volcano plot showing differentially expressed genes (DEGs) in OVCAR3 cells cultured on Col1 + WRG-28 compared with cells cultured on Col1 alone. DEGs from four independent experiments were identified based on statistical significance (FDR < 0.05 and |log2 FC| > 0.58) and are highlighted in red. Vertical dashed lines indicate FC thresholds, and horizontal dashed line FDR significance threshold. Arrows indicate the number of significantly upregulated and downregulated DEGs. Red boxes highlight selected ECM-associated genes (MMP1, MMP7, MMP10, SPARC, FAP, and COL14A1). (**B**) Gene Ontology (GO) cellular component enrichment analysis of significantly downregulated (DN) DEGs. Upregulated genes do not show significant enriched categories.

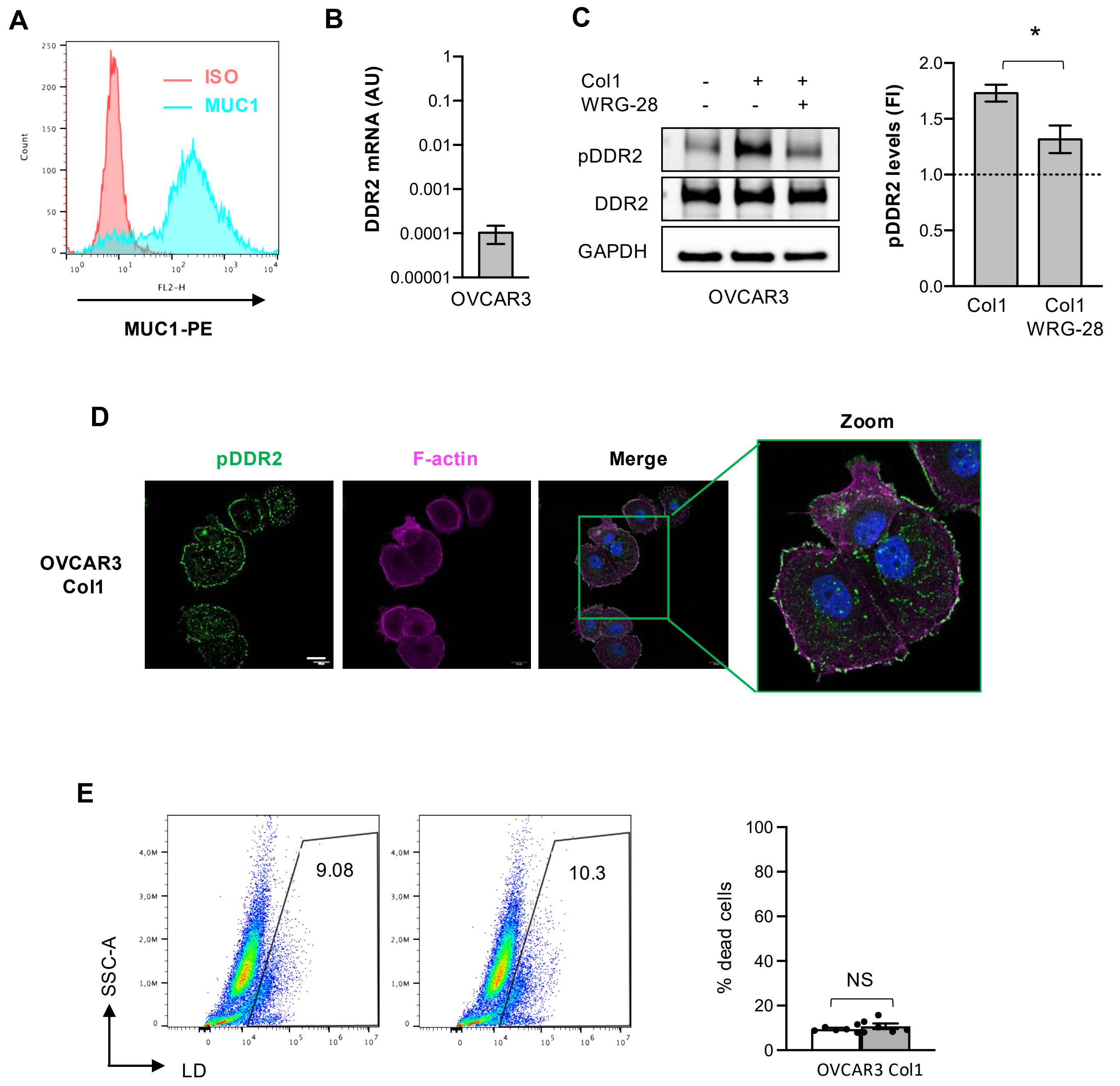

Fig. S4. Immunophenotype of OVCAR3 cells. (**A**) Flow cytometry analysis of MUC1 surface expression in OVCAR3 cells. ISO, isotype control antibodies. (**B**) Real-time PCR analysis of DDR2 mRNA levels in OVCAR3 cells, normalised to GAPDH and expressed as arbitrary units (AU). Data represent the mean ± SEM from four independent experiments. (**C**) Representative western blot analysis of phosphorylated DDR2 (pDDR2), total DDR2, and GAPDH in OVCAR3 cells cultured on Col1 in the presence of DMSO (vehicle control) or 2 µM WRG-28. Fold induction (FI) of pDDR2 levels relative to Ctr was calculated after normalisation to GAPDH levels. Data represent the mean ± SEM from three independent experiments. (**D**) Representative fluorescence microscopy images of pDDR2 (green) in OVCAR3 cells cultured on Col1 for 6 hours. Nuclei were stained with DAPI (blue), and F-actin with phalloidin (magenta). Scale bar = 25 µm. (**E**) Live/dead (LD) flow cytometry analysis of OVCAR3 cells cultured for 24 hours on Col1 in the presence of DMSO (vehicle control, Ctr) or WRG-28. Data represent the mean percentage of dead cells ± SEM from six independent experiments. Statistical significance was determined by Student's t-test. * $p < 0.05$. NS = not significant.

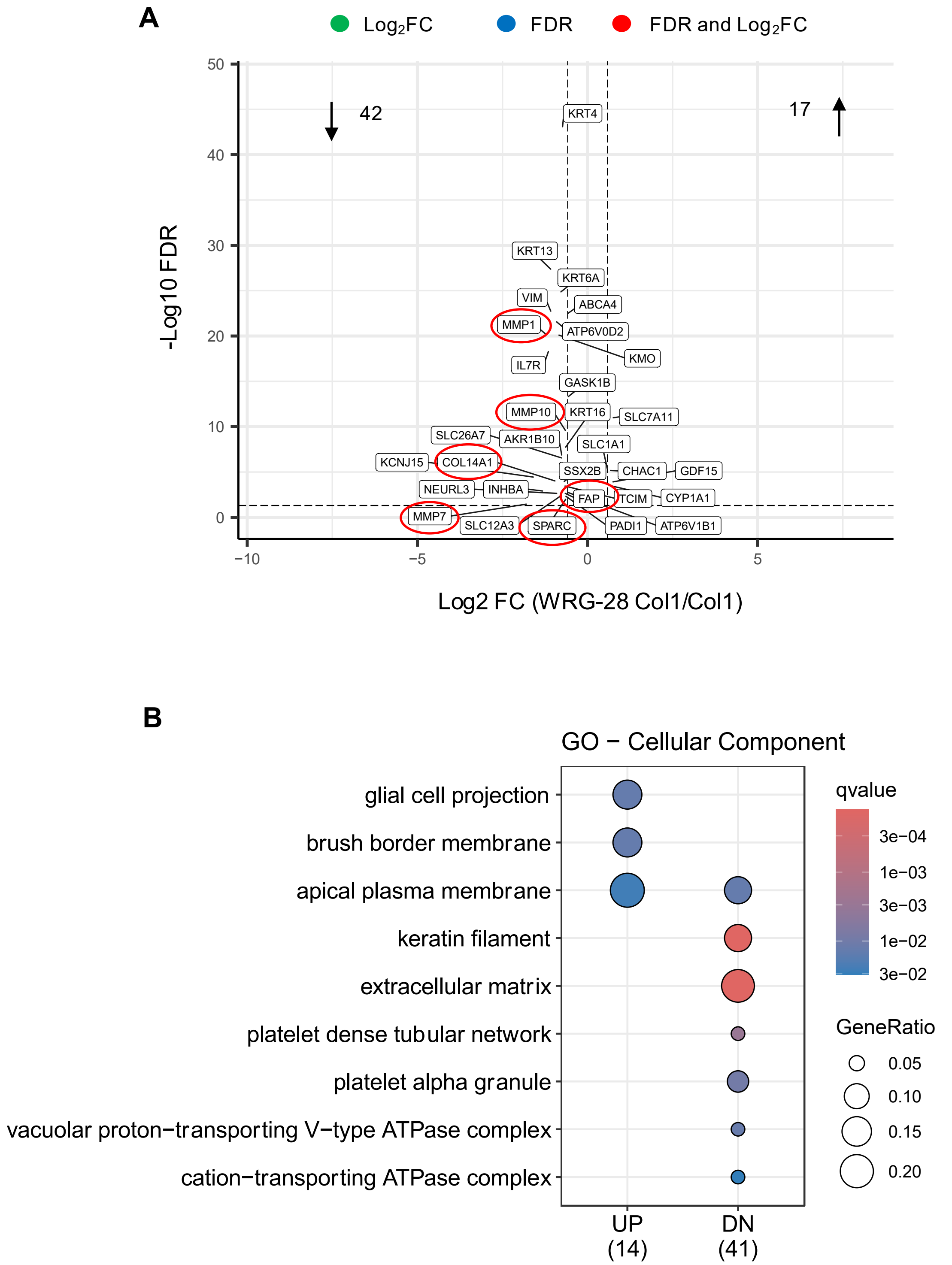

Fig. S5. Transcriptomic profiling of OVCAR3 cells. (**A–B**) RNA-seq analysis of OVCAR3 cells cultured on Col1 in the presence of DMSO (vehicle control) or 2 μM WRG-28 for 24 hours. (**A**) Volcano plot showing differentially expressed genes (DEGs) in OVCAR3 cells cultured on Col1 + WRG-28 compared with cells cultured on Col1 alone. DEGs from four independent experiments were identified based on statistical significance (FDR < 0.05 and |log2 FC| > 0.58) and are highlighted in red. Vertical dashed lines indicate FC thresholds, and horizontal dashed line indicates the FDR significance threshold. Arrows indicate the number of significantly upregulated and downregulated DEGs. Red boxes highlight selected ECM-associated genes (MMP1, MMP7, MMP10, SPARC, FAP, and COL14A1). (**B**) Gene Ontology (GO) cellular component enrichment analysis of significantly upregulated (UP) and downregulated (DN) DEGs.

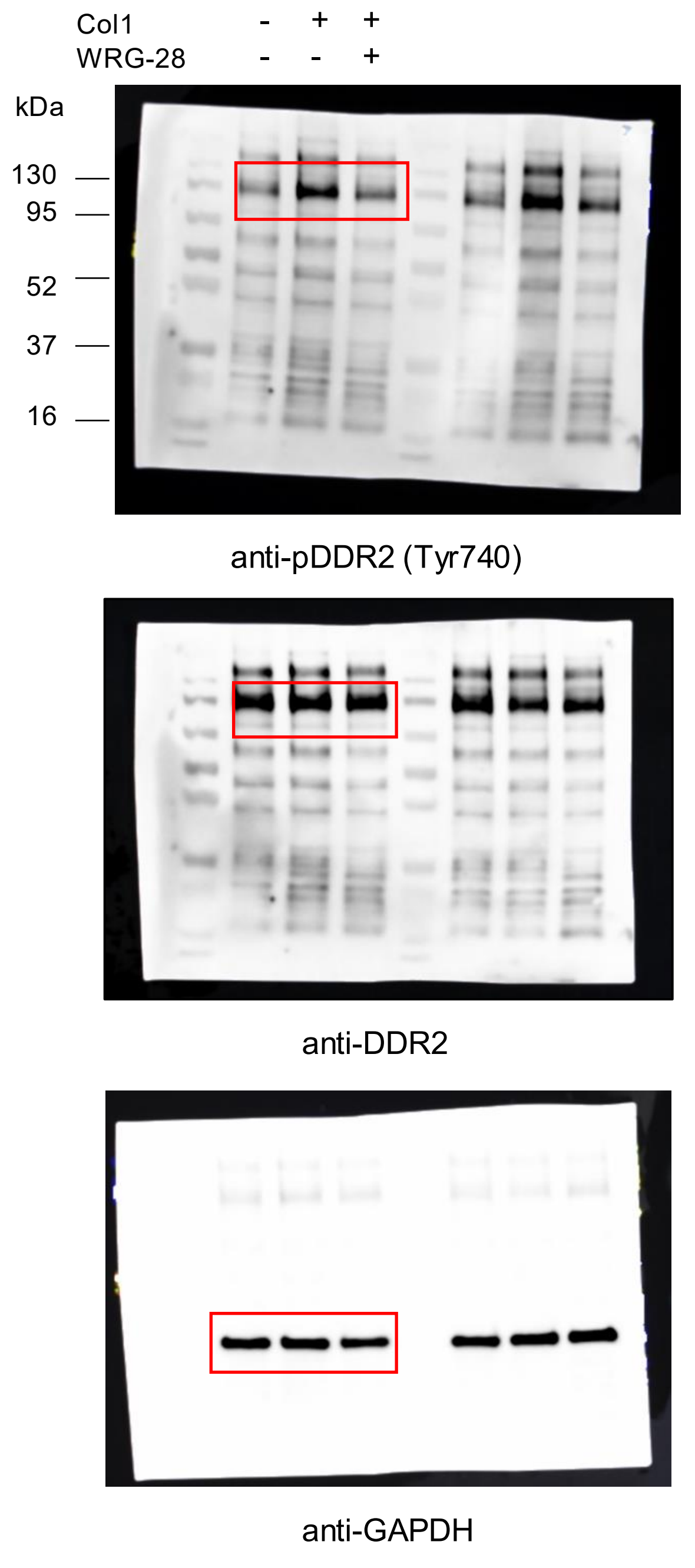

Fig. S6. Original immunoblot data corresponding to Additional Fig.S4C. Molecular weight markers are indicated in kDa.

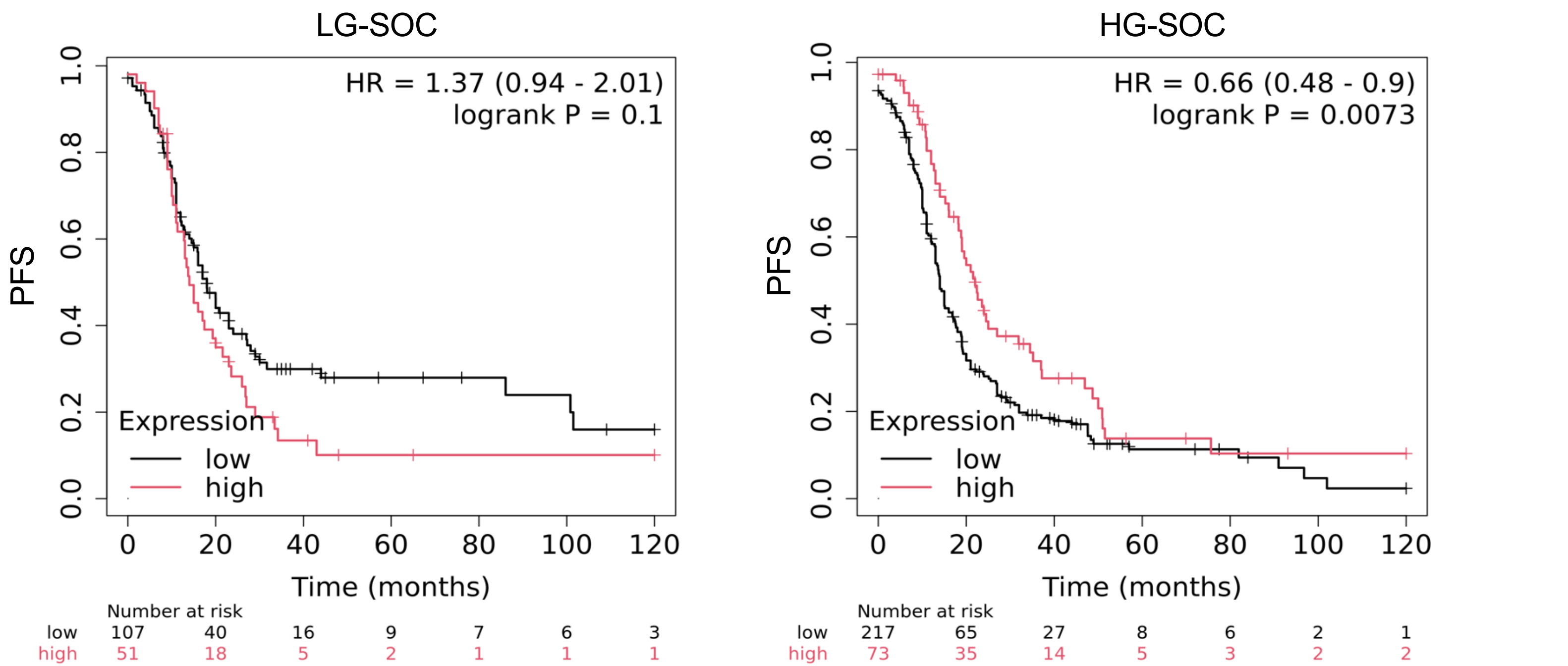

Fig. S7. Prognostic impact of DDR2/MUC1 co-expression in SOC. Kaplan–Meier analysis of progression-free survival (PFS) according to DDR2 and MUC1 co-expression levels in LG-SOC and HG-SOC. High DDR2 expression is associated with reduced PFS in HG-SOC. Statistical significance was determined using the log-rank test.
