# Supplementary material for: Boosting ovarian cancer immunotherapy via discoidin domain receptor 2 blockade and CD28 x mucin 1 bispecific antibodies
Source: BMC Med. 2026 Aug 24;24:598. doi: 10.1186/s12916-026-05183-2 (PMC13721643; doi:10.1186/s12916-026-05183-2)
Supplement: Supplementary file 2 [file 12916_2026_5183_MOESM5_ESM.pdf]

Additional files

The online version contains additional files available at

Additional file 1: Table 1. Information on antibodies used in multicolour flow cytometry.

Additional file 2: Table 2. Transcriptomic profiling of OVCA433 following WRG-28 treatment.

Additional file 3: Table 3. Transcriptomic profiling of OVCAR3 following WRG-28 treatment.

Additional file 4: Figure S1-S7. Fig S1-DDR2 expression in OVCA433 and TOVC cells. Fig S2-Phenotypic characterization of CD8-enriched TOVC cell lines. Fig S3-Transcriptomic profiling of OVCA433 cells. Fig S4-Immunophenotype of OVCAR3 cells. Fig S5-Transcriptomic profiling of OVCAR3 cells. Fig S6-Original immunoblot data corresponding to Additional file 4: Fig.S4C. Fig S7-Prognostic impact of DDR2/MUC1 co-expression in SOC.
